# Seminal Microbiota of Idiopathic Infertile Patients and Its Relationship With Sperm DNA Integrity

**DOI:** 10.3389/fcell.2022.937157

**Published:** 2022-06-28

**Authors:** Sergio Garcia-Segura, Javier del Rey, Laia Closa, Iris Garcia-Martínez, Carlos Hobeich, Ana Belén Castel, Francisco Vidal, Jordi Benet, Jordi Ribas-Maynou, Maria Oliver-Bonet

**Affiliations:** ^1^ Unit of Cell Biology and Medical Genetics, Department of Cell Biology, Physiology and Immunology, Universitat Autònoma de Barcelona (UAB), Bellaterra, Spain; ^2^ Histocompatibility and Immunogenetics Laboratory, Banc de Sang i Teixits (BST), Barcelona, Spain; ^3^ Medicina Transfusional, Vall d’Hebron Institut de Recerca (VHIR), Universitat Autònoma de Barcelona (UAB), Barcelona, Spain; ^4^ Grup de Coagulopaties Congènites, Banc de Sang i Teixits (BST), Barcelona, Spain; ^5^ Instituto de Fertilidad, Palma, Spain; ^6^ CIBER de Enfermedades Cardiovasculares (CIBERCV), Barcelona, Spain; ^7^ Biotechnology of Animal and Human Reproduction (TechnoSperm), Institute of Food and Agricultural Technology, University of Girona, Girona, Spain; ^8^ Unit of Cell Biology, Department of Biology, University of Girona, Girona, Spain

**Keywords:** seminal microbiome, human fertility, male infertility, sperm dna damage, oxidative stress, next generation sequencing

## Abstract

The development of new biomarkers for human male infertility is crucial to improve the diagnosis and the prognosis of this disease. Recently, seminal microbiota was shown to be related to sperm quality parameters, suggesting an effect in human fertility and postulating it as a biomarker candidate. However, its relationship to sperm DNA integrity has not been studied yet. The aim of the present study is to characterize the seminal microbiota of a western Mediterranean population and to evaluate its relationship to sperm chromatin integrity parameters, and oxidative stress. For that purpose, 14 samples from sperm donors and 42 samples from infertile idiopathic patients were obtained and were analyzed to assess the composition of the microbiota through full-length *16S rRNA* gene sequencing (Illumina MiSeq platform). Microbial diversity and relative abundances were compared to classic sperm quality parameters (macroscopic semen parameters, motility, morphology and concentration), chromatin integrity (global DNA damage, double-stranded DNA breaks and DNA protamination status) and oxidative stress levels (oxidation-reduction potential). The seminal microbiota observed of these samples belonged to the phyla *Firmicutes*, *Proteobacteria*, *Actinobacteria* and *Bacteroidetes*. The most abundant genera were *Finegoldia*, *Peptoniphilus*, *Anaerococcus*, *Campylobacter*, *Streptococcus*, *Staphylococcus*, *Moraxella*, *Prevotella*, *Ezakiella*, *Corynebacterium* and *Lactobacillus*. To our knowledge, this is the first detection of *Ezakiella* genus in seminal samples. Two clusters of microbial profiles were built based on a clustering analysis, and specific genera were found with different frequencies in relation to seminal quality defects. The abundances of several bacteria negatively correlate with the sperm global DNA fragmentation, most notably *Moraxella*, *Brevundimonas* and *Flavobacterium*. The latter two were also associated with higher sperm motility and *Brevundimonas* additionally with lower oxidative-reduction potential. *Actinomycetaceae*, *Ralstonia* and *Paenibacillus* correlated with reduced chromatin protamination status and increased double-stranded DNA fragmentation. These effects on DNA integrity coincide in many cases with the metabolism or enzymatic activities of these genera. Significant differences between fertile and infertile men were found in the relative presence of the *Propionibacteriaceae* family and the *Cutibacterium*, *Rhodopseudomonas* and *Oligotropha* genera, which supports its possible involvement in male fertility. Our findings sustain the hypothesis that the seminal microbiome has an effect on male fertility.

## Introduction

Infertility is a multiorganism and multifactorial disease affecting millions of humans worldwide. The male counterpart is known to exert an impact in about 50% of couples who fail to conceive after a year of regular unprotected sexual intercourse (de Kretser, 1997; Boivin et al., 2007; Agarwal et al., 2015). Nowadays, diagnostic assessment of male infertility is rather limited, with only the assessment of sperm motility, sperm count or sperm morphology parameters used universally. However, about 25% of infertile men present values within the normal range for these parameters and are thus considered normozoospermic. In these cases, infertility causes usually remain undiagnosed (Poongothai et al., 2009). Therefore, finding new biomarkers for idiopathic male infertility is key to advancing our knowledge of the disease. Biomarkers would, not only lay the groundwork for the development of new therapeutic strategies, but also serve as diagnostic and prognostic tools with predictive value in *in vitro* fertilization methodologies (Brincat et al., 2015). In an attempt to address this need, several fertility biomarkers have been studied over the last decade (Kovac et al., 2013; Boissière et al., 2017; Martins and Agarwal, 2019; Mehrparavar et al., 2019). Among them, the measurement of oxidative stress and sperm DNA integrity have provided valuable information related to the balance of the seminal redox system and the effect of reactive oxygen species (ROS) on chromatin integrity and other sperm structures such as plasma and mitochondrial membranes (Aitken and De Iuliis, 2010; Ribas-Maynou and Benet, 2019; Agarwal et al., 2020). In this regard, different authors have shown that increased ROS levels and the incidence of DNA damage are factors intimately related to male infertility (Lewis and Simon, 2010; Aitken et al., 2014; Pourmasumi et al., 2017; Agarwal et al., 2019). However, the association of these biomarkers to success rates after intracytoplasmic sperm injection are still controversial (Ribas-Maynou et al., 2021).

Recent studies focused on different human diseases support the essential role of the microbiome in health-disease status (Shreiner et al., 2015; Young, 2017; Giles and Couper, 2020). The development of Next Generation Sequencing (NGS) techniques has allowed a deeper analysis of the microbiomes colonizing humans (Malla et al., 2019) and, for this reason, the number of studies analyzing the microbiomes’ composition and relation to health and diseases has grown exponentially over the last decade (Laudes et al., 2021). Similarly, seminal microbiota dysbiosis has recently been proposed as a potential cause of male infertility (Altmäe et al., 2019; Koedooder et al., 2019; Lundy et al., 2020; Tomaiuolo et al., 2020; Farahani et al., 2021). However, the number of published studies addressing this association is still limited and results show discrepancies regarding microbiome composition, which suggests a very high interindividual variability. Although no definitive consensus has been reached as yet, several authors agree that seminal microbiota is composed of the phyla *Firmicutes, Bacteroidetes, Proteobacteria* and *Actinobacteria* (Lundy et al., 2020; Tomaiuolo et al., 2020; Farahani et al., 2021). Specifically, the most commonly identified genera are *Lactobacillus*, *Corynebacterium*, *Acinetobacter*, *Prevotella*, *Enterococcus*, *Veillonella*, *Streptococcus*, *Porphyromonas*, *Staphylococcus* and *Pelomonas* (Hou et al., 2013; Weng et al., 2014; Mändar et al., 2017; Chen et al., 2018; Monteiro et al., 2018; Baud et al., 2019; Amato et al., 2020; Yang et al., 2020; Bukharin et al., 2021; Lundy et al., 2021; Okwelogu et al., 2021; Yao et al., 2021).

The lack of agreement on the role of the seminal microbiome on male fertility is manifold. Some authors report an increase of species diversity in infertile men’s semen (Rehewy et al., 1979; Mändar et al., 2017; Amato et al., 2020; Lundy et al., 2021), while others do not find differences between clinical groups (Jarvi et al., 1996; Hou et al., 2013; Weng et al., 2014; Yang et al., 2020). Several studies have found that *Lactobacillus*, frequently described as the most abundant genus in semen, is less present in men with decreased seminal quality, and it is especially related to motility and viscosity parameters (Weng et al., 2014; Mändar et al., 2017; Monteiro et al., 2018; Baud et al., 2019). However, other studies have reported opposite results, showing an increase of this genus (and others such as *Ureaplasma*, *Finegoldia* and *Anaerococcus*) in asthenozoospermic and azoospermic patients (Hou et al., 2013; Chen et al., 2018; Yang et al., 2020). A higher consensus exists regarding the genus *Prevotella*, which has always been associated with a decrease in sperm quality, mainly underlain by a reduction in sperm motility (Weng et al., 2014; Baud et al., 2019; Yang et al., 2020; Lundy et al., 2021). Finally, some genera of *Proteobacteria* and *Actinobacteria* have also been directly related to seminal hyperviscosity, oligoasthenoteratozoospermia and azoospermia (Weng et al., 2014; Chen et al., 2018; Monteiro et al., 2018; Yang et al., 2020).

Despite the growing evidence supporting the association between the seminal microbiome and conventional sperm quality parameters, no studies assessing the impact of seminal microbiota on sperm chromatin and oxidative stress have been published so far. Therefore, the aim of the present study was to analyze the seminal microbiota of a western Mediterranean population and evaluate its relationship with sperm chromatin status, oxidative stress and sperm quality.

## Materials and Methods

### Sample Collection

Seminal samples were collected from 56 caucasian Mediterranean subjects, which were classified into two groups: 14 control samples from healthy normozoospermic semen donors with no infertility diagnosis and 42 samples from idiopathic normozoospermic infertile patients with no apparent female factor as a possible cause of the couple’s infertility. Individuals under antibiotic therapy for the last 21 days were excluded. Demographic and health information is shown in Supplementary Table S1. A specific semen collection protocol adapted from the World Health Organization (WHO) procedures (World Health Organization, 2010), which included urinating and washing hands and penis with soap and water before masturbation into a sterile container, was followed to prevent bacterial contamination of samples. Environmental samples were also collected from collection room by moving a sterile swab in the air. A seminogram was performed on samples from all groups according to the WHO 2010 guidelines (World Health Organization, 2010).

Samples were obtained after 2–5 days of sexual abstinence from patients attending the *Instituto de Fertilidad* of Palma (Mallorca, Spain). After liquefaction at room temperature, samples were aliquoted and frozen at −20°C without cryopreservation medium, transported to the laboratory and stored in liquid nitrogen at −196°C until further use. Informed consent was obtained from all donors, and the study was approved by the Parc Taulí Hospital ethics committee according to the Declaration of Helsinki.

### Conventional Sperm Quality Assessment: Concentration, Motility, Morphology, pH and Viscosity

Prior to cryopreservation, a basic semen analysis (sperm concentration, motility, morphology, seminal volume and pH) was performed. For concentration and motility, 7 μl of prewarmed (37°C) and liquefied sperm were loaded into a prewarmed 10 micron Leja Chamber (Leja Products B.V., Luzemestraat, Nieuw Vennep, Netherlands). Then, videos were taken at 30 frames per second using the Sperm Class Analyzer (SCA, Microptic, Barcelona, Spain) software coupled to a negative phase contrast microscope (Olympus Provis AX70, Olympus Corporation, Tokio, JP). For each sample, at least 200 spermatozoa in at least five homogeneous fields were evaluated, and the following parameters were recorded: sperm concentration, percentage of motile sperm, percentage of progressively motile sperm, percentage of non-progressive motile sperm and percentage of immotile sperm. For morphology, Sperm Blue (Microptic, Barcelona, Spain) was used to stain sperm cells. Briefly, 10–15 μl were poured onto the slides, covered with a 22 × 22 mm coverslip and visualized under a morphology module of the SCA software on a brightfield microscope (Olympus Provis AX70). The percentage of abnormal sperm was assessed using Kruger strict criteria, recording alterations in head, midpiece, tail and cytoplasmic droplets. PH indicator strips (MColorpHast, Merck, Darmstadt, Germany) were used to determine the sample pH. Seminal viscosity was measured using 20 micron Leja Chambers (Leja Products B.V.), following a protocol previously described by Rijnders et al., 2007. Briefly, a standard curve relating viscosity and time to fill the chamber was generated using solutions with different known viscosity. Seminal viscosity was then measured by calculating the sample’s capillary filling time and results were reported in centipoise (cps).

### Oxidative Stress Evaluation (MiOXSYS)

Seminal oxidative stress was assessed by oxidation-reduction potential (ORP) measurement using the MiOXSYS system (Aytu BioScience, Englewood, CO, United States) and following the manufacturer’s instructions. Briefly, 30 μl were loaded onto a cassette and inserted into the apparatus, which provided a relative value indicating static oxidation-reduction potential. Two replicates were conducted per sample, and the obtained average was normalized by sperm concentration (mV/10^6^ sperm/ml) to obtain normalized static oxidation-reduction potential (nsORP).

### Sperm Chromatin Evaluation

#### Sperm DNA Integrity

Global sperm DNA fragmentation analysis, including single- and double-stranded DNA breaks, was performed by the TUNEL and Alkaline Comet assays. Double-stranded DNA breaks were also analyzed by Neutral Comet.

For the TUNEL assay, the *In Situ* Cell Death Detection Kit (Roche Diagnostic GmbH, Penzberg, Germany) was used, following the manufacturer’s instructions. Semen samples were washed three times in phosphate buffered saline solution (PBS) before adjusting the sperm concentration to 5 × 10^6^ cells/ml. Resuspended sperm samples were divided into three 200 µl tubes (positive control, negative control and test sample), fixed with 4% paraformaldehyde in PBS for 1 h at room temperature and then washed in PBS supplemented with 1% bovine serum albumin (BSA; Sigma-Aldrich, St. Louis, MO, United States). Sperm cells were permeabilized using 0.1% Triton X-100 in 0.1% sodium citrate for 2 min in ice and then washed in PBS supplemented with 1% BSA. A positive control was prepared by incubating the sample in 100 IU DNAse I (Roche Diagnostic GmbH) for 10 min at 37°C while the test sample and the negative control were kept on ice. The positive control and test sample pellets were then incubated in 45 µl of labelling solution plus 5 µl of terminal deoxynucleotidyl transferase (TdT) enzyme (Roche Diagnostic GmbH) for 1 h at 37°C in the dark, while the negative control followed the same procedure, with the omission of the TdT enzyme. Finally, samples were washed twice using 1% BSA in PBS. Pellets were resuspended in 1 ml of PBS and analyzed by flow cytometry. In order to detect the TUNEL-positive cells (TUNEL^+^; green fluorescent cells), samples were excited with a blue laser (488 nm) and the emitted fluorescence was collected using a 530 ± 30 nm band-pass filter. A total of 10,000 events at 200–300 cells/s were recorded on a flow cytometer (FACSCalibur; Becton Dickinson, NJ, United States), and data were processed using CELLQUEST analysis software (Becton Dickinson). Negative and positive controls were used to establish the threshold values for TUNEL^+^ cells and, finally, the percentage of TUNEL^+^ cells was recorded.

The Comet assay was performed in both alkaline and neutral conditions, according to previously described methodology (Casanovas et al., 2019). First, samples were washed and adjusted to 1 × 10^6^ sperm/ml. Sperm cells were mixed with low-melting-point 1% agarose in a 1:2 proportion to achieve a final agarose concentration of 0.66%. Then, a drop of the mix was allowed to gel at 4°C on an agarose-pretreated slide and an 8-mm round coverslip was applied. Sperm nucleus decondensation was effected by incubating the slides in two consecutive lysis solutions: lysis solution one, containing 0.8 M DTT, 0.8 M Tris, 1% SDS, pH = 7.5, for 30 min, and lysis solution two, containing 0.4 M DTT, 0.4 M Tris, 2 M NaCl, 50 mM EDTA, 1% Tween20, pH 7.5, for 30 min. Subsequently, for the Alkaline Comet assay slide, a denaturing treatment in an alkaline solution (0.03 M NaOH and 1 M NaCl) for 2.5 min at 4°C was performed, followed by electrophoresis at 1 V/Cm for 4 min in 0.03 M NaOH buffer. In parallel, for the Neutral Comet assay slide, electrophoresis was performed for 12.5 min at 1 V/Cm in TBE buffer with a subsequent wash in 0.9% NaCl solution. Finally, both slides were washed in a neutralization solution (0.04 M Tris-HCl, pH 7.5), dehydrated in ethanol series (70, 90 and 100%) and dried horizontally before staining with DAPI SlowFade Gold anti-fade (Invitrogen, Carlsbad, CA, United States). Samples were analyzed and evaluated under a fluorescence microscope by a single researcher to avoid intra-observer variability. Fragmented and non-fragmented spermatozoa were manually classified, using criteria reported earlier (Ribas-Maynou et al., 2012), and the percentage of sperm cells with DNA fragmentation was recorded. To assess and quantify the incidence of DNA breaks, both alkaline and neutral Comets were captured under the same epifluorescence microscope and analyzed using Komet 7 analysis software (Andor Technologies, Oxford, United Kingdom). The Olive Tail Moment (OTM), calculated as [(Tail mean intensity–Head mean intensity) × Tail DNA/100] was recorded for each Comet variant as an indicator of DNA damage intensity, in arbitrary units.

#### Sperm Chromatin Protamination Status (Chromomycin A_3_ Test)

Sperm chromatin protamination was assessed using the Chromomycin A_3_ (CMA_3_, Sigma-Aldrich) test. Briefly, semen samples were washed twice in PBS, permeabilized with 0.5% Triton X-100, and resuspended to a sperm concentration of 10^7^ cells/ml. Sperm cells were subsequently spread on a slide and air-dried. The slide was then incubated with 50 µl of a staining solution containing 250 μg/ml CMA_3_, 10 mM MgCl_2_, 30 mmol/L citric acid, 140 mmol/L Na_2_HPO_4_ at room temperature for 20 min in the dark and covered with Parafilm. Finally, the Parafilm was gently removed and sperm cells were counterstained with DAPI SlowFade Gold antifade (Invitrogen), and a coverslip was applied. A total of 400 cells was analyzed under an epifluorescence microscope and evaluated to obtain the percentage of CMA_3_ positive cells, as described previously (Garcia-Segura et al., 2020).

### Microbiome Analysis

#### Microbiota DNA Extraction

Bacterial DNA isolation from seminal samples was performed with ZymoBIOMICS DNA Microprep kit (Zymo Research, CA, EEUU) following the manufacturer’s instructions with slight modifications. In order to lyse cell walls and membranes to allow the release of DNA, a bead beating step in lysis solution was performed. Then, all supernatant was filtered in Zymo-Spin III-F by centrifugation at 8,000 g for 1 min and washing repeatedly in a Zymo-Spin IC-Z column to purify DNA before elution. The researcher worked with sterile gloves in a horizontal laminar flow cabinet previously sterilized with DNA-degrading products and UV irradiation. A sterile swab was placed inside the cabinet as a negative environmental control and the negative blanks of extraction were also sequenced to observe the *kitome*.

#### *16S rRNA* Gene Sequencing

To characterize seminal microbiota, all nine hypervariable regions of *16S rRNA* gene (V1-V9) were sequenced. The amplicons of the V1-V9 *16S rRNA* gene regions were obtained by two consecutive PCRs. The universal *16S rRNA* primers 27F and 1492R (Lane, 1991) were used in the first PCR with the addition of a specific tag (27F: 5′-TTTCTGTTGGTGCTGATATTGCAGRGTTTGATYHTGGCTCAG-3′; 1492R: 5′-ACTTGCCTGTCGCTCTATCTTCTACCTTGTTAYGACTT-3′, tag underlined). The PCR Mix contained 10.3 µl nuclease-free water, 4 µl 5X buffer, 2 µl 2 mM dNTP, 0.8 µl 10 µM forward primer, 1.6 µl 10 µM reverse primer, 0.3 µl 2 U/µl Phusion Hot Start II Taq HIFI polymerase (Thermo Fisher Scientific; Waltham MA, United States) and 1 µl DNA template per sample. The reactions were carried out in an Veriti Thermal Cycler (Applied Biosystems, Thermo Fisher Scientific) and set as follows: initial denaturation at 98°C for 30 s, followed by 25 cycles of denaturation at 98°C for 15 s, annealing at 62.5°C for 15 s and extension at 72°C for 45 s. The program was completed with a final extension at 72°C for 7 min.

The second PCR was performed using SequalPrep polymerase (Thermo Fisher Scientific) with self-designed primers that targeted the specific tag incorporated in the first PCR. PCR mix per sample was as follows: 4.67 µl nuclease-free water, 1 µl 10X buffer, 0.55 µl 5.5% DMSO, 1 µl 10X Enhancer, 0.1 µl 50 mM MgCl2, 1.5 µl 10 µM primer mix, 0.18 µl 5 U/ml SequalPrep polymerase and 1 µl of a 1:10 dilution of the first PCR’s DNA product. The thermocycler was set with an initial denaturation at 94°C for 60 s, followed by 20 cycles of denaturation at 94°C for 30 s, annealing at 62°C for 30 s and extension at 65°C for 75 s. The program ended with a final extension at 65°C for 5 min. Negative PCR blanks products from both amplifications were pooled and sequenced as a *kitome* control for PCR reagents.

DNA library preparation was performed by enzymatic fragmentation of the PCR product and double indexing using the NGSgo kit (GenDx, Utrecht, Netherlands), according to the GENDX NGSgo workflow (2017, 4th edition). The indexed libraries were pooled, denatured, and diluted to a final concentration of 4 nM. Pooled libraries were sequenced on the MiSeq system (Illumina, San Diego, CA, United States), according to the manufacturer’s instructions.

#### Bioinformatics Analysis

The quality of paired-end sequencing data in FASTQ file format was evaluated with the CLC Genomics Workbench software (Qiagen, Hilden, Germany). Subsequently, sequencing data were processed with the FastQC Toolkit (BaseSpace, Illumina) and reads were descarted based on the following criteria: read length <140 bp and Q-Phred score <30. Taxonomic classification of bacterial reads and alpha diversity (Shannon index) of each sample was performed following the DRAGEN Metagenomics pipeline (v3.5) (BaseSpace, Illumina), which uses the Kraken2 algorithm (v2.0) and the corresponding Extended Kraken2 taxonomic database (March 2020) (Wood and Salzberg, 2014). Prior to analysis, human reads were dehosted from each sample using the same application (UCSC HG19Alt-Aware). Raw relative abundances at phyla, families and genera taxonomic levels were formatted using the R environment. Dataset obtained from sequencing and associated metadata, with the results of seminal quality analyses, is available on-line (Garcia-Segura et al., 2022) in the Dipòsit Digital de Documents (DDD) of Universitat Autònoma de Barcelona.

### Statistical Analyses

Statistical analyses were conducted using the IBM SPSS Statistics 25.0 software (IBM Corp., Armonk, NY, United States), and graphs were generated in GraphPad Prism v.8 (GraphPad Software, La Jolla, CA, United States). First, statistical normality of quantitative seminal parameters was checked using the Shapiro-Wilk test. If parametric assumptions were not fitted even after linear transformation (arcsin √x, √x), non-parametric assumptions were necessary to analyze the results. Correlations between quantitative variables were assessed through the non-parametric Spearman test. Differences of microbiological relative abundance (phyla, families and genera) between donors and infertile patients, as well as between samples with altered/normal oxidative stress or chromatin damage, were assessed using the Mann-Whitney *U* test. Differences regarding the presence or absence of a particular microorganism between clinical conditions, oxidative stress status or presence of chromatin damage were assessed using the Chi-squared test.

To investigate the presence of distinct microbiologic profiles at the phyla, families or genera taxonomic levels, a cluster analysis was conducted considering the relative abundances of identified bacteria in the whole sample, using the between-groups linkage method based on the Euclidean distance. Clustering for families and genera was tested using only taxa with >1% of abundance. Clustering analysis was performed regardless of fertility status of samples. Differences in sperm quality quantitative parameters and alpha diversity between individuals assigned to each cluster were compared using the Mann-Whitney *U* test, and fertility condition was compared using the Chi-squared test. For those genera with a significant association to fertility status, a Principal Component Analysis (PCA) was run to sort the genera into a single component. The obtained data was rotated with the Varimax procedure (Kaiser, 1959) and a variable with a loading factor higher than 0.6 and lower than 0.3 in the rotated matrix was selected. The component was used to calculate a regression score for each sample to identify the predictive value for male infertility. For all statistical tests, the level of significance was set at 95% of the confidence interval (*p* < 0.05).

## Results

### Seminal Microbiota Composition

After applying the quality criteria, the sequencing of the *16S rRNA* in the study samples generated an average of 221,542.88 reads per sample with a mean length of 146.76 bp and an average depth of 21,517.51 defined theoretically as LN/G, where L is the read length, N is the number of reads and G is the gene length (Sims et al., 2014). The percentage of reads that exceeded the established Q-Phred 30 was 87.99%. In contrast, contamination controls (sample collection, DNA extraction and PCR controls) displayed 10-fold fewer DNA copies, with an average of 6,321 reads. Due to this low reads count in the contamination controls, no contamination of the study samples was assumed, so no correction of the relative abundances of the detected bacteria was applied. Bacterial profiles observed in contamination controls are shown in Supplementary Figure S1.

**FIGURE 1:**
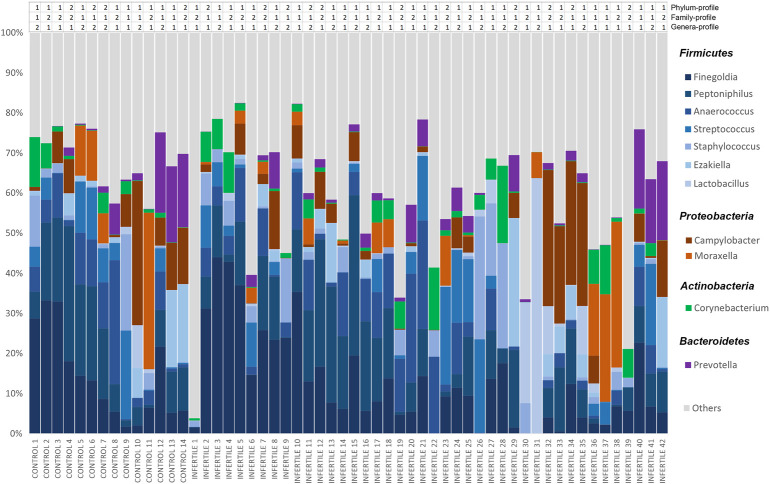
Relative abundances of bacteria included in seminal microbiome from control donors and idiopathic infertile patients at genus level. The *X*-axis shows each individual of our cohort, and the *Y*-axis corresponds to the relative abundance of each taxon in percentage. Clustering-based bacterial profiles to which each individual belongs is shown above the bar-graph at phylum, family and genera levels.

Considering the whole sample, seminal microbiota was mainly composed of four phyla: *Firmicutes* (∼59%), *Proteobacteria* (∼19%), *Actinobacteria* (∼8%) and *Bacteroidetes* (∼5%). A total of 168 genera were identified, among which the most abundant were *Finegoldia*, *Peptoniphilus*, *Anaerococcus*, *Campylobacter*, *Streptococcus*, *Staphylococcus*, *Moraxella*, *Prevotella*, *Ezakiella*, *Corynebacterium* and *Lactobacillus* (Figure 1). These 11 genera accounted for 75.24% of all reads classified in the Bacteria Kingdom, and the least represented genus showed an abundance of >3%. At the family level, *Peptostreptococcaceae* and *Veillonellaceae* were also found among the most abundant, along with the families to which the previous genera belonged (Supplementary Table S2). Of note, these results highlight the great interindividual variability among the relative abundances of these taxa (Figure 1).

### Seminal Microbiota Structure: Bacterial Profiles and Diversity

**FIGURE 2:**
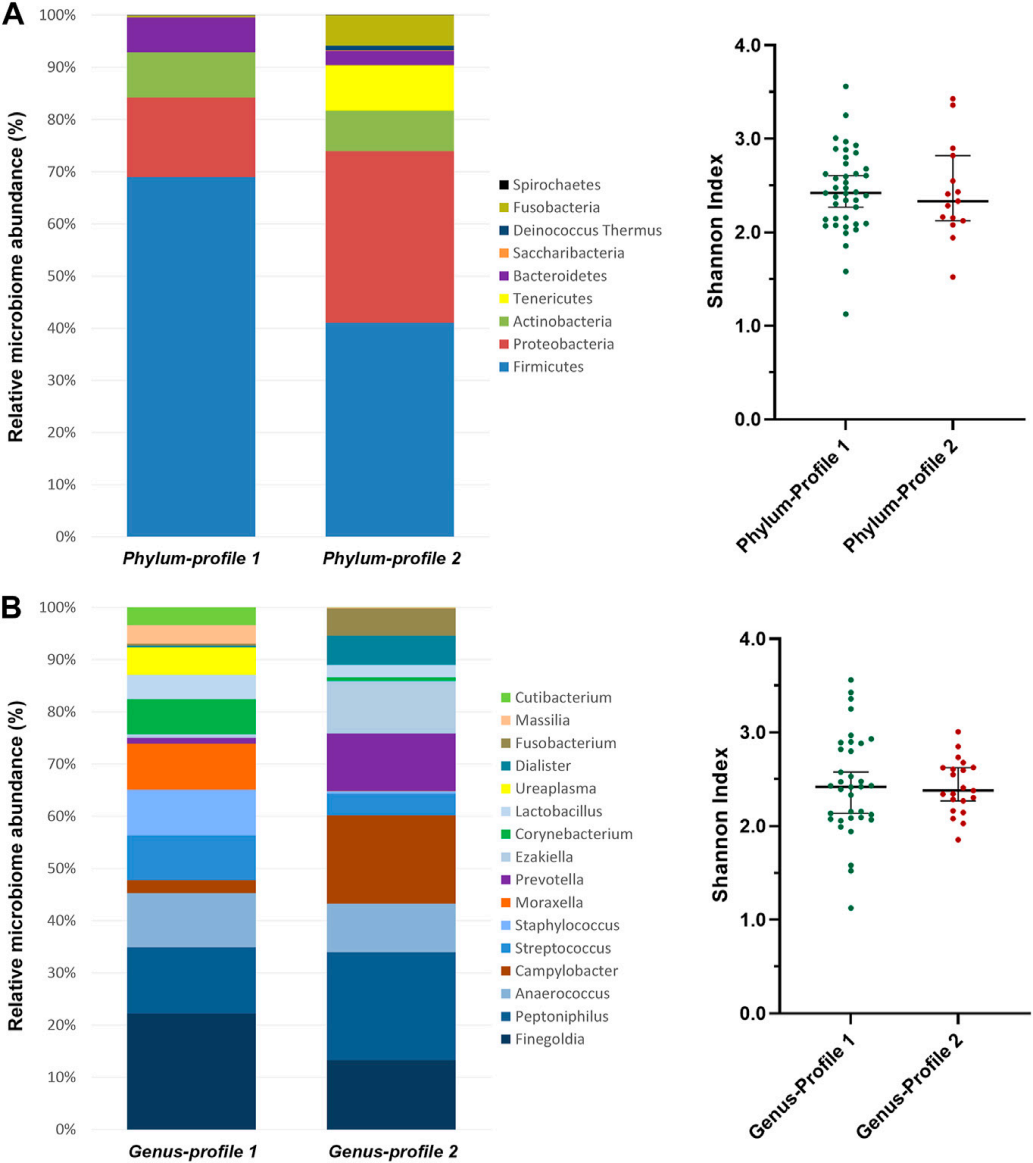
Microbiome profiles’ composition from clustering analysis of **(A)** phylum and **(B)** genus taxonomic levels. Clustering analysis was performed using the between-groups linkage method based on the Euclidean distance. The bar-plots show the relative abundance in percentage of the most representative bacteria of each profile. The scatter dot-plots display the alpha diversity distribution of each profile, where the *Y*-axis represents the Shannon index. Thin horizontal lines delimit the 95% confidence interval (CI), whereas the thick horizontal mark denotes the median value.

Two bacterial profiles were identified for each taxonomical level (phylum, family and genus) by clustering analysis of whole sample (Supplementary Table S3). At the phylum level, phylum-profile 1 was dominated mainly by *Firmicutes* while phylum-profile 2 maintained similar frequencies of *Firmicutes* and *Proteobacteria*, with the presence of *Tenericutes* and *Fusobacteria* as well (Figure 2A). However, the alpha diversity was not found significantly different between these clusters (*p* = 0.760).

At the family-level, *Peptoniphilaceae* was predominant in both clusters, but more abundant in the family-profile 1. Family-profile 1 was characterized by the presence of *Campylobacteraceae*, *Prevotellaceae*, *Peptostreptococcaceae*, *Veillonellaceae*, *Fusobacteriaceae* and *Actinomycetaceae*, and family-profile 2 comprised *Moraxellaceae*, *Staphylococcaceae*, *Corynebacteriaceae*, *Lactobacillaceae*, *Mycoplasmaceae*, *Oxalobacteraceae*, *Propionibacteriaceae* and *Burkholderiaceae* (Supplementary Table S3). As for the phylum level, no significant differences in alpha diversity were found between these clusters (*p =* 0.088).

At the genus-level, *Finegoldia* was the most abundant in profile 1, followed by *Staphylococcus*, *Moraxella*, *Corynebacterium*, *Ureaplasma*, *Massilia* and *Cutibacterium*. In contrast, *Peptoniphilus* and *Campylobacter* predominated in profile 2, shared with *Prevotella*, *Ezakiella*, *Dialister* and *Fusobacterium*. Genus-profile 1 showed an enrichment of *Streptococcus* and *Lactobacillus* when compared to genus-profile 2 (Figure 2B). As in the previous clusterings, alpha diversity was not different between the two clusters (*p =* 0.980).

Association analysis between microbiome profiles and different clinical phenotypes retrieved no significant results (*p >* 0.05). Patients and controls were evenly distributed between the two observed clusters at all taxa levels (Table 1). Similarly, no significant differences in conventional sperm quality parameters, chromatin status or oxidative stress were found between the microbiome profile 1 and 2 (*p >* 0.05) (Table 2).

**TABLE 1:** Distribution of infertile patients and donors between the two microbiome profiles revealed by the clustering analysis based on the bacterial relative abundance both at the phylum and genus taxonomic levels, using the between-groups linkage method based on the Euclidean distance.

	Phylum level		Genus level	
	Cluster 1	Cluster 2	Cluster 1	Cluster 2
Donors	10	4	8	6
Infertile	31	11	27	15
*p*-value	0.862		0.633	

The *p* value for the association analysis with the Pearson’s Chi-square test is shown. The threshold for statistical significance was set at *p* < 0.05.

**TABLE 2:** Comparative study of seminal and sperm parameters (Mann-Whitney’s U) between microbiome profile 1 (P1) and profile 2 (P2) at phylum and genera levels.

	Phylum level			Genera level		
	P1 (*n* = 41)	P2 (*n* = 15)	*p*-value	P1 (*n* = 35)	P2 (*n* = 21)	*p*-value
Volume (ml)	2.93	2.35	0.134	2.90	2.70	0.662
pH	8.23	8.15	0.555	8.20	8.24	0.617
Viscosity (cps)	5.75	5.35	0.483	5.63	5.59	0.626
Sperm concentration (M sperm/ml)	59.00	88.60	0.295	76.00	54.45	0.399
Sperm total number (×10^6)	199.80	174.00	0.961	177.30	160.50	0.714
Total motility (%)	49.50	62.00	0.09	49.17	61.00	0.204
Progressive motility (%)	32.66	49.58	0.221	38.00	34.99	0.978
Sperm morphology (%)	8.80	9.50	0.235	9.75	8.25	0.298
Normed sORP (mV/10^6^ sperm/ml)	0.74	0.38	0.179	0.60	0.53	0.546
CMA3^+^ cells (%)	39.00	39.04	0.858	39.00	39.04	0.759
TUNEL^+^ cells (%)	40.46	41.30	0.685	40.75	41.79	0.879
Alkaline comet (%)	38.00	33.00	0.63	34.33	39.00	0.298
Alkaline OTM	0.87	0.80	0.767	0.86	0.86	0.431
Neutral comet (%)	65.66	65.00	0.882	65.33	66.00	0.472
Neutral OTM	0.61	0.58	0.218	0.60	0.58	0.509

Median of each parameter for each cluster is shown. The threshold for statistical significance was set at *p* < 0.05.

**FIGURE 3:**
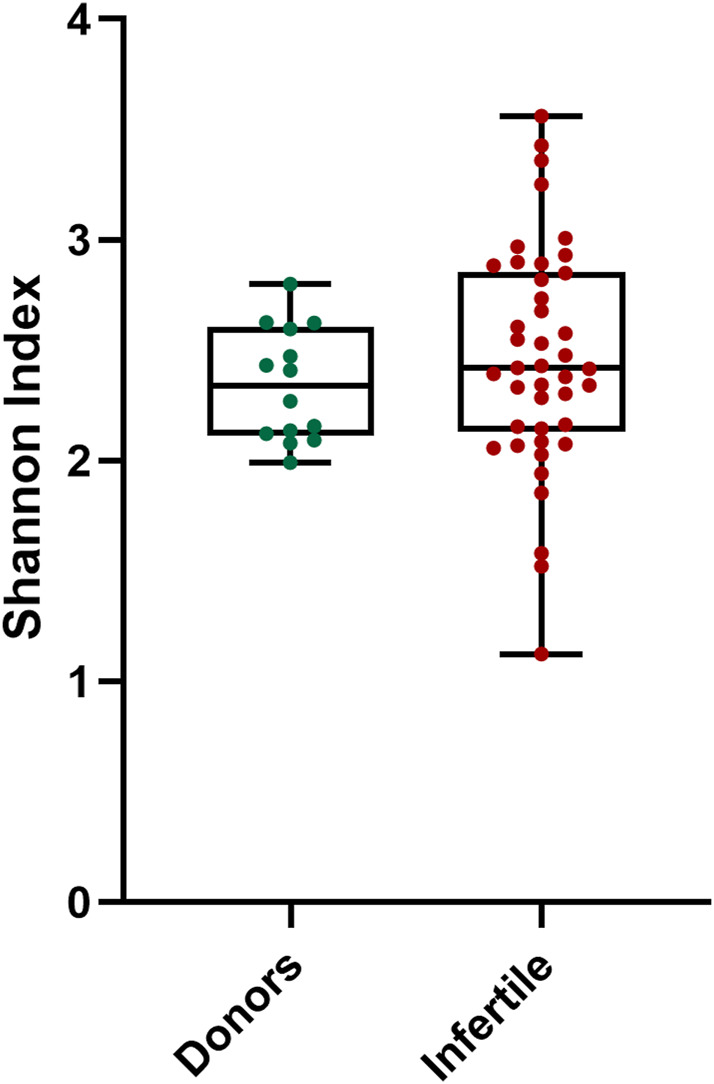
Alpha diversity distribution for microbiota of donors and infertile patients. *Y*-axis represents the Shannon index of the groups displayed at the *X*-axis. Thin horizontal lines delimit the 95% confidence interval (CI), whereas the thick horizontal mark denotes the median value.

The correlation analysis between alpha diversity and seminal quality parameters in all samples revealed a significant association between higher alpha diversity and increased percentage of sperm with double-stranded DNA fragmentation (Neutral Comet assay) (Table 3). However, no significant differences in alpha diversity were observed between infertile patients and the control group (Figure 3).

**TABLE 3:** Results from the correlation analyses between alpha diversity and seminal and sperm parameters.

Seminal or sperm parameter (units)	Spearman ρ	*p*-value
Seminal volume (ml)	-0.056	0.686
pH	0.198	0.147
Seminal viscosity (cps)	-0.037	0.79
Sperm concentration (M/ml)	0.106	0.45
Total sperm number (×10^6^)	0.072	0.607
Total motility (%)	-0.018	0.897
Progressive motility (%)	0.037	0.795
Sperm morphology (%)	0.089	0.567
Normed sORP (mV/10^6^sperm/ml)	0.08	0.575
CMA3^+^ cells (%)	-0.003	0.983
TUNEL^+^ cells (%)	-0.146	0.292
Alkaline comet (%)	-0.143	0.292
Alkaline OTM	-0.064	0.642
Neutral comet (%)	**0.320**	**0.016**
Neutral OTM	0.23	0.089

The Spearman Rho (ρ) and the associated *p*-value are shown. The threshold for statistical significance was set at *p* < 0.05. Values in bold indicate nominally significant associations.

### Impact of Seminal Microbiota on Conventional Semen Quality Parameters

Significant correlations were found between the relative abundances of bacterial taxa and some seminogram parameters, in all samples of our cohort regardless the fertility status (Table 4). Increased seminal volume was found associated with higher abundance of *Ralstonia*, *Bacillus* and *Steroidobacter*, and lower presence of *Janibacter*. The abundance of the *Leptotrichiaceae* and *Neisseriaceae* families (specifically, the *Neisseria* genus) was found to increase with higher seminal pH. Lower seminal viscosity was associated with an increase of the *Comamonadaceae*, *Pasteurellaceae* and *Promicromonosporaceae* (especially, the *Xylanomonas* genus) families, as well as of the *Ralstonia*, *Schaalia*, *Aerococcus* and *Megasphaera* genera.

**TABLE 4:** Correlation coefficients for the abundance of identified (A) phylum, (B) family or (C) genera and basic sperm quality parameters.

A *PHYLUM*	Semen volume (ml)	Sperm concentration (×10^6)	Total motile sperm (%)	Progressive motility (%)	Normal morphology (%)	pH	Semen viscosity (cps)
Firmicutes	0.067	−0.165	0.019	−0.118	−0.183	0.097	−0.163
Proteobacteria	−0.155	0.064	−0.093	−0.062	0.255	0.027	0.124
Actinobacteria	0.035	0.084	0.020	0.225	0.016	−0.201	0.046
Tenericutes	−0.101	0.079	−0.049	0.074	−0.241	−0.106	−0.019
Bacteroidetes	−0.003	−0.041	0.160	0.100	−0.020	0.106	−0.003
Saccharibacteria	0.022	−0.035	−0.099	−0.031	−0.158	0.160	0.057
Deinococcus.Thermus	−0.098	−0.114	0.107	0.112	−0.243	0.187	0.018
Fusobacteria	−0.206	0.084	−0.019	−0.109	0.124	0.136	−0.168
Spirochaetes	0.065	−0.054	−0.128	−0.209	−0.090	0.095	−0.249

B *FAMILY*	Semen volume (ml)	Sperm concentration (×10^6)	Total motile sperm (%)	Progressive motility (%)	Normal morphology (%)	pH	Semen viscosity (cps)
Peptoniphilaceae	−0.046	−0.069	−0.073	−0.176	0.062	0.027	−0.168
Campylobacteraceae	−0.172	0.012	−0.085	−0.193	−0.139	−0.078	−0.089
Streptococcaceae	0.077	0.035	−0.059	−0.047	0.110	0.171	−0.257
Moraxellaceae	0.047	0.112	−0.166	−0.029	0.351*	0.032	0.026
Staphylococcaceae	0.086	0.094	−0.05	0.093	−0.057	−0.249	−0.088
Prevotellaceae	−0.058	−0.091	0.101	0.004	0.052	0.199	0.045
Corynebacteriaceae	0.041	0.089	−0.133	0.007	0.062	−0.214	−0.161
Peptostreptococcaceae	−0.088	−0.071	−0.004	−0.085	0.037	0.093	−0.053
Lactobacillaceae	−0.025	0.013	0.196	0.252	−0.167	0.011	−0.097
Veillonellaceae	−0.083	−0.047	0.051	−0.064	−0.024	0.031	−0.173
Mycoplasmataceae	−0.038	0.027	−0.199	−0.097	−0.141	−0.185	−0.179
Oxalobacteraceae	−0.085	0.1	−0.113	−0.051	0.374*	0.015	−0.126
Propionibacteriaceae	0.092	−0.156	0.097	0.290*	0.039	−0.052	0.089
Fusobacteriaceae	−0.236	0.164	0.013	−0.102	0.209	0.090	−0.118
Actinomycetaceae	−0.043	−0.057	0.114	−0.009	−0.120	0.037	−0.172
Burkholderiaceae	0.156	−0.21	0.021	−0.058	−0.026	0.227	−0.061
Bradyrhizobiaceae	0.184	−0.151	−0.033	0.088	−0.064	−0.051	0.005
Rhodobacteraceae	0.061	0.119	0.125	0.005	0.215	0.059	−0.023
Caulobacteraceae	−0.003	0.165	0.196	0.331*	−0.013	−0.157	−0.053
Bacillaceae	0.245	−0.117	0.109	0.003	−0.111	−0.035	−0.228
Porphyromonadaceae	−0.044	−0.002	0.023	0.024	−0.163	0.026	−0.122
Sphingomonadaceae	0.022	−0.062	−0.291*	−0.232	−0.045	0.128	−0.064
Bifidobacteriaceae	0.09	−0.013	0.293*	0.362**	−0.326*	0.001	−0.19
Aerococcaceae	0.027	−0.024	0.097	0.027	−0.198	0.179	−0.167
Clostridiaceae	−0.079	−0.179	0.227	0.049	−0.137	0.082	−0.031
Flavobacteriaceae	−0.064	−0.032	0.107	0.173	−0.173	−0.101	−0.14
Neisseriaceae	−0.098	−0.088	−0.062	−0.106	−0.019	0.345**	−0.198
Deinococcaceae	−0.112	−0.095	0.195	0.179	−0.384*	0.042	−0.1
Paenibacillaceae	0.245	−0.336*	0.023	−0.01	−0.202	0.080	−0.053
Bacteroidaceae	−0.049	−0.075	0.098	0.058	−0.091	0.046	−0.066
Micrococcaceae	−0.016	−0.019	0.064	0.033	0.040	0.068	−0.19
Lachnospiraceae	−0.027	−0.341*	0.074	0.1	−0.293	0.214	−0.071
Comamonadaceae	0.163	0.01	−0.032	0.01	0.030	−0.050	−0.386**
Enterobacteriaceae	0.162	−0.154	−0.039	0.073	−0.081	−0.031	−0.021
Intrasporangiaceae	−0.204	0.024	0.067	0.045	0.137	−0.201	−0.095
Thermoanaerobacteraceae	−0.082	−0.197	0.015	0.082	−0.140	−0.005	0.016
Pasteurellaceae	0.029	−0.189	0.035	0.104	−0.285	0.029	−0.309*
Promicromonosporaceae	0.158	−0.164	−0.048	−0.039	−0.055	−0.148	−0.325*
Methylobacteriaceae	0.038	−0.043	0.046	−0.093	0.145	−0.033	−0.205
Listeriaceae	0.157	0.033	0.09	−0.045	−0.022	0.152	−0.158
Erysipelotrichaceae	0.003	−0.038	0.201	0.102	−0.036	0.143	−0.106
Hungateiclostridiaceae	−0.196	0.089	0.151	0.006	−0.143	0.104	−0.006
Tissierellaceae	−0.035	−0.134	0.031	−0.056	−0.088	−0.160	−0.013
Steroidobacteraceae	0.256	−0.019	0.133	0.11	−0.053	0.237	−0.051
Acidiferrobacteraceae	0.199	−0.03	0.108	0.079	0.082	0.205	0.092
Erwiniaceae	−0.084	−0.209	−0.038	−0.133	−0.360*	0.056	0.006
Enterococcaceae	−0.07	0.015	−0.063	−0.085	−0.079	−0.125	−0.15
Spirochaetaceae	0.077	−0.079	−0.127	−0.238	−0.092	0.086	−0.198
Sinobacteraceae	0.182	−0.087	0.027	0.047	−0.007	0.227	0.012
Alteromonadaceae	−0.142	−0.01	−0.056	−0.235	−0.049	−0.109	0.036
Thermoanaerobacterales.Family.III..Incertae.Sedis	−0.078	−0.127	0.037	−0.16	−0.195	0.107	−0.013
Leptotrichiaceae	−0.167	−0.094	0.003	−0.021	−0.134	0.291*	−0.053
Atopobiaceae	0.051	−0.076	0.093	0.069	0.030	−0.033	−0.11
Piscirickettsiaceae	0.218	−0.098	0.147	0.126	−0.069	0.170	0.141
**C *GENERA***	**Semen volume (ml)**	**Sperm concentration (×10^6)**	**Total motile sperm (%)**	**Proggressive motility (%)**	**Normal morphology (%)**	**pH**	**Semen viscosity (cps)**
Finegoldia	0.127	−0.159	−0.088	−0.144	0.045	−0.099	−0.268
Peptoniphilus	−0.190	0.049	−0.147	−0.306*	−0.018	0.127	0.005
Anaerococcus	−0.110	0.011	0.028	0.044	0.253	0.224	−0.094
Campylobacter	−0.127	0.017	−0.086	−0.203	−0.111	−0.073	−0.083
Streptococcus	0.104	0.035	−0.064	−0.050	0.113	0.170	−0.256
Staphylococcus	0.067	0.104	−0.044	0.095	−0.057	−0.245	−0.09
Moraxella	−0.092	0.142	−0.045	0.085	0.357*	−0.006	0.049
Prevotella	−0.026	−0.106	0.090	−0.008	0.043	0.196	0.032
Ezakiella	−0.071	−0.036	−0.062	−0.243	−0.053	−0.051	0.047
Corynebacterium	0.027	0.077	−0.142	0.006	0.036	−0.214	−0.152
Lactobacillus	−0.026	0.027	0.214	0.258	−0.142	0.021	−0.08
Ureaplasma	−0.110	0.153	−0.102	−0.063	0.272	−0.179	−0.119
Dialister	−0.006	−0.065	0.078	−0.016	−0.044	−0.054	−0.084
Fusobacterium	−0.187	0.170	0.013	−0.107	0.221	0.084	−0.132
Massilia	−0.110	0.083	−0.108	−0.035	0.337*	−0.004	−0.143
Cutibacterium	0.118	−0.078	−0.046	0.189	0.231	−0.162	−0.006
Ralstonia	0.307*	−0.322*	0.047	−0.032	−0.116	0.168	−**0**.346*
Veillonella	−0.093	0.150	0.094	−0.059	0.119	0.106	−0.005
Mobiluncus	−0.182	−0.188	0.082	0.003	−0.109	−0.091	−0.013
Parvimonas	−0.138	0.002	−0.090	−0.117	−0.019	0.000	−0.055
Murdochiella	0.070	−0.241	−0.094	−0.112	−0.230	−0.110	−0.007
Acinetobacter	0.132	−0.085	−0.127	−0.062	0.237	0.070	−0.156
Gemella	−0.069	0.017	−0.026	−0.037	−0.325*	0.085	−0.159
Porphyromonas	−0.003	−0.006	0.031	0.020	−0.160	0.039	−0.118
Brevundimonas	−0.088	0.244	0.118	0.266*	0.067	−0.140	−0.034
Paracoccus	−0.110	0.047	0.104	0.096	0.184	0.049	0.035
Gardnerella	−0.153	0.024	0.227	0.289*	−0.417**	0.028	−0.002
Clostridium	−0.040	−0.168	0.251	0.082	−0.140	0.054	0.004
Actinomyces	0.121	−0.179	0.154	0.052	−0.221	−0.012	−0.237
Deinococcus	−0.081	−0.095	0.195	0.179	−0.384*	0.042	−0.1
Bacillus	0.284*	−0.114	0.061	−0.025	−0.104	−0.030	−0.172
Sphingobium	−0.064	−0.130	−0.172	−0.215	0.067	0.214	−0.101
Suicoccus	−0.016	0.025	0.113	0.034	−0.016	0.240	0.08
Bradyrhizobium	0.117	−0.153	−0.028	0.020	−0.084	−0.060	−0.096
Bacteroides	−0.015	−0.075	0.098	0.058	−0.091	0.046	−0.066
Schaalia	−0.050	0.014	−0.033	−0.122	0.118	0.089	−0.289*
Neisseria	−0.093	−0.065	−0.081	−0.105	0.001	0.332*	−0.143
Paenibacillus	0.249	−0.328*	−0.048	−0.073	−0.228	0.089	−0.034
Aerococcus	0.051	−0.118	0.185	0.135	−0.262	−0.058	−0.290*
Actinotignum	0.025	0.122	0.118	−0.105	0.052	−0.065	−0.01
Flavobacterium	−0.206	0.100	0.243	0.292*	−0.119	−0.163	−0.159
Sphingomonas	−0.100	0.075	0.081	0.050	0.007	0.075	−0.115
Cupriavidus	0.089	−0.109	−0.021	−0.016	0.075	0.115	0.003
Fermentimonas	−0.189	0.110	−0.083	−0.071	−0.160	−0.103	−0.163
Xylanimonas	0.131	−0.164	−0.048	−0.039	−0.055	−0.148	−0.325*
Janibacter	−0.266*	0.107	0.038	0.030	0.290	−0.024	−0.162
Tepidanaerobacter	0.001	−0.258	−0.039	0.003	−0.149	0.002	−0.045
Bifidobacterium	0.249	−0.149	0.138	0.157	−0.119	0.009	−0.137
Kocuria	0.027	0.044	−0.162	−0.095	0.243	−0.097	−0.141
Propionimicrobium	−0.025	−0.057	0.108	−0.013	−0.083	0.145	0.037
Megasphaera	0.094	−0.231	0.093	−0.041	−0.129	−0.082	−0.356**
Steroidobacter	0.272*	−0.019	0.133	0.110	−0.053	0.237	−0.051
Brochothrix	0.092	−0.103	0.180	0.220	−0.039	0.115	−0.111
Rhodopseudomonas	0.008	−0.170	0.021	0.207	−0.110	−0.004	0.08
Haemophilus	−0.112	−0.134	−0.077	−0.080	−0.221	0.096	−0.183
Enterococcus	−0.033	0.040	−0.048	−0.101	−0.023	−0.216	−0.204
Oligotropha	0.037	−0.028	0.047	0.205	0.024	−0.062	0.076
Glaciecola	−0.125	0.004	0.004	−0.219	−0.022	−0.052	0.105
Filifactor	0.042	−0.090	0.369**	0.256	−0.124	0.102	−0.093
Thermoanaerobacterium	−0.061	−0.135	0.051	−0.137	−0.187	0.099	0.022

Labeled in bold are the statistically significant correlations. *Statistically significant correlations (*p* < 0.05) ** Statistically significant correlations (*p* < 0.01).

Decreased sperm concentration was found to correlate with higher abundance of the *Paenibacillaceae* (specifically *Paenibacillus*) and *Lachnospiraceae* families, and the *Ralstonia* genus. Sperm morphology was found associated with an increased presence of *Moraxella* and *Massilia*, and a decreased presence of the *Deinococcaceae* family and the *Deinococcus* and *Gardnerella* genera. Finally, total motility was found to increase with the presence of the *Filifactor* genus, as well as with the family *Bifidobacteriaceae* and its genus *Gardnerella*. This genus showed an even more intense association with increased progressive motility. Likewise, the *Propionibacteriaceae* and *Caulobacteraceae* families and the *Flavobacterium* genus were associated with higher progressive motility, whereas the presence of the *Peptoniphilus* genus correlated with a decreased progressive motility. Finally, the presence of *Sphingomonadaceae* family also correlated with a decreased total motility.

### Impact of Microbiome on Oxidative Stress and Sperm Chromatin

Table 5 shows correlation analysis results between the relative abundance of bacterial taxa, oxidative stress and sperm chromatin parameters, regardless the fertility status. Increased oxidative stress, assessed as higher nsORP, was found to correlate with higher abundance of *Oligotropha*, *Rhodopseudomonas* (both genera of the *Bradyrhizobiaceae* family), *Megasphaera* and *Paenibacillus* (also with *Paenibacillaceae* family). On the other hand, the *Brevundimonas* genus was associated with lower nsORP. An increased abundance of *Sphingomonadaceae* (especially with its genus *Sphingomonas*) and *Schaalia* was associated with sperm chromatin compaction alterations, whereas the abundance of the *Paenibacillaceae* family, specifically *Paenibacillus*, and the *Bacillus* and *Ralstonia* genera correlated to better protamination status.

**TABLE 5:** Correlation coefficients for the abundance of identified phylum, family or genera and oxidative stress and chromatin status.

A *PHYLUM*	Normalized sORP	CMA_3_^+^ cells (%)	TUNEL^+^ (%)	Global DNA breaks intensity (alkaline comet olive tail moment)	Percentage of fragmented cells (% alkaline comet)	Double strand DNA breaks intensity (neutral comet olive tail moment)	Percentage of fragmented cells (% neutral comet)
Firmicutes	0.114	−0.014	0.233	0.233	0.2	0.022	−0.177
Proteobacteria	−0.028	−0.098	−0.143	−0.382**	−0.226	0.136	0.015
Actinobacteria	−0.069	−0.13	0.099	0.053	−0.137	−0.022	−0.046
Tenericutes	−0.09	0.072	0.118	−0.182	−0.254	−0.141	0.204
Bacteroidetes	0.047	−0.004	−0.193	−0.018	−0.042	−0.025	0.203
Saccharibacteria	0.019	0.083	−0.001	−0.109	−0.156	0.04	0.093
Deinococcus.Thermus	0	0.122	0.193	0.194	0.217	−0.17	−0.103
Fusobacteria	−0.09	0.055	−0.106	−0.104	0.004	0.066	0.027
Spirochaetes	0.159	0.183	0.059	0.088	0.196	0.031	−0.045

B *FAMILY*	Normalized sORP	Sperm with poor protamination CMA3+ (%)	Sperm with DNA breaks TUNEL+ (%)	DNA breaks intensity (Alkaline Comet Olive Tail Moment)	Percentage of fragmented cells (%Alkaline Comet)	Double strand DNA breaks intensity (Neutral Comet Olive Tail Moment)	Percentage of fragmented cells (% Neutral Comet)
Peptoniphilaceae	0.026	0.035	0.057	0.155	0.263	0.105	−0.158
Campylobacteraceae	0.046	0.093	0.003	0.094	0.101	−0.12	0.089
Streptococcaceae	−0.143	−0.056	0.158	0.189	0.06	−0.056	−0.147
Moraxellaceae	−0.093	−0.014	−0.041	−0.248	−0.288*	0.189	−0.004
Staphylococcaceae	−0.135	−0.033	−0.019	0.039	−0.02	0.016	0.039
Prevotellaceae	0.073	0.143	−0.182	0.091	0.079	0.069	0.173
Corynebacteriaceae	−0.156	0.081	0.203	0.225	0.03	−0.047	−0.146
Peptostreptococcaceae	0.061	0.223	0.185	0.134	0.238	−0.153	−0.12
Lactobacillaceae	−0.03	−0.175	0.089	−0.242	−0.285*	−0.041	0.115
Veillonellaceae	0.122	0.026	−0.055	0.075	−0.015	0.041	0.207
Mycoplasmataceae	−0.071	0.069	0.135	−0.078	−0.17	−0.105	0.227
Oxalobacteraceae	−0.081	−0.227	−0.092	−0.2	−0.192	0.259	0.074
Propionibacteriaceae	0.191	−0.229	−0.177	−0.2	−0.178	0.074	0.149
Fusobacteriaceae	−0.141	0.035	−0.119	−0.128	−0.045	0.016	0.007
Actinomycetaceae	−0.014	0.107	0.061	0.153	0.041	0.197	0.084
Burkholderiaceae	0.055	−0.142	0.024	−0.07	0.004	0.378**	0.144
Bradyrhizobiaceae	0.252	−0.178	−0.163	0	0.066	−0.011	−0.067
Rhodobacteraceae	−0.003	0.048	−0.011	−0.317*	−0.256	0.242	0.015
Caulobacteraceae	−0.167	−0.042	−0.376**	−0.155	−0.207	0.021	0.154
Bacillaceae	0.161	−0.259	0.128	0.193	0.039	0.047	−0.004
Porphyromonadaceae	0.064	−0.046	0.015	0.001	−0.134	−0.129	0.164
Sphingomonadaceae	0.128	0.301*	−0.011	−0.124	−0.053	0.075	0.06
Bifidobacteriaceae	0.093	−0.178	0.079	−0.173	−0.136	0.075	0.17
Aerococcaceae	0.001	−0.042	0.188	−0.004	−0.023	−0.093	−0.078
Clostridiaceae	0.11	0.037	0.06	0.153	0.135	0.012	0.024
Flavobacteriaceae	−0.018	−0.15	−0.16	−0.089	−0.136	−0.038	0.117
Neisseriaceae	0.003	0.228	0.04	−0.116	−0.058	−0.018	−0.011
Deinococcaceae	−0.045	0.027	0.176	0.17	0.179	−0.151	0.002
Paenibacillaceae	0.323*	−0.390**	−0.121	0.2	−0.037	0.26	0.290*
Bacteroidaceae	0.131	−0.049	−0.085	0.054	−0.021	−0.133	0.147
Micrococcaceae	0.076	0.058	0.11	−0.19	−0.231	0.016	0.04
Lachnospiraceae	0.235	−0.102	−0.039	−0.07	0.043	0.228	0.239
Comamonadaceae	0.167	−0.071	−0.12	−0.248	−0.166	0.264*	0.165
Enterobacteriaceae	0.082	0.006	−0.082	0.013	−0.017	−0.114	−0.021
Intrasporangiaceae	0.032	−0.049	−0.07	0.027	−0.033	−0.2	0.057
Thermoanaerobacteraceae	0.142	0	0.104	0.158	0.294*	−0.005	−0.025
Pasteurellaceae	0.213	0.078	−0.05	−0.004	−0.01	−0.008	0.163
Promicromonosporaceae	0.242	−0.142	0.007	0.212	0.151	−0.181	−0.178
Methylobacteriaceae	0.15	−0.014	0.11	0.018	−0.036	0.158	0.053
Listeriaceae	−0.207	0.046	0.049	0.009	−0.077	0.017	0.102
Erysipelotrichaceae	0.114	0.047	0.07	−0.026	0.012	−0.013	0.056
Hungateiclostridiaceae	−0.039	0.186	0.143	0.063	0.127	−0.083	0.14
Tissierellaceae	0.182	−0.045	0.027	0.086	0.085	0.068	0.152
Steroidobacteraceae	0.016	0.012	−0.011	−0.192	−0.143	0.172	−0.085
Acidiferrobacteraceae	−0.025	0.045	0.096	−0.184	−0.108	0.006	−0.214
Erwiniaceae	0.191	0.135	0.1	0.018	0.144	−0.154	0.012
Enterococcaceae	0.136	−0.005	−0.005	0.246	0.127	0.228	0.304*
Spirochaetaceae	0.141	0.189	0.063	0.108	0.225	−0.024	−0.096
Sinobacteraceae	0.043	0.01	0.128	−0.081	−0.045	0.058	−0.236
Alteromonadaceae	0.079	0.03	−0.052	0.033	0.023	−0.041	0.167
Thermoanaerobacterales.Family.III..Incertae.Sedis	0.095	0.105	0.085	0.221	0.218	0.114	0.116
Leptotrichiaceae	−0.046	0.219	−0.037	0.151	0.156	0.024	0.176
Atopobiaceae	0.014	0.084	0.003	0.015	0.134	−0.038	0.054
Piscirickettsiaceae	0.068	0.166	0.095	−0.099	−0.043	−0.093	−0.098

C *GENERA*	Normalized sORP	Sperm with poor protamination CMA3+ (%)	Sperm with DNA breaks TUNEL+ (%)	DNA breaks intensity (Alkaline Comet Olive Tail Moment)	Percentage of fragmented cells (%Alkaline Comet)	Double strand DNA breaks intensity (Neutral Comet Olive Tail Moment)	Percentage of fragmented cells (% Neutral Comet)
Finegoldia	0.053	−0.007	0.043	0.088	0.133	0.087	−0.196
Peptoniphilus	−0.016	0.166	0.088	0.046	0.142	0.23	−0.025
Anaerococcus	0.058	0.086	0.019	−0.017	0.206	0.074	−0.117
Campylobacter	0.047	0.119	0.014	0.082	0.109	−0.126	0.071
Streptococcus	−0.146	−0.053	0.156	0.196	0.067	−0.061	−0.153
Staphylococcus	−0.148	−0.02	−0.01	0.035	−0.024	0.01	0.036
Moraxella	−0.075	0.018	−0.045	−0.319*	−0.343**	0.104	−0.033
Prevotella	0.085	0.131	−0.181	0.084	0.07	0.073	0.176
Ezakiella	0.03	0.031	−0.039	0.06	0.123	−0.072	0.04
Corynebacterium	−0.143	0.083	0.204	0.227	0.027	−0.058	−0.144
Lactobacillus	−0.04	−0.157	0.083	−0.252	−0.280*	−0.052	0.115
Ureaplasma	−0.206	0.064	−0.113	0.006	−0.117	−0.178	0.094
Dialister	0.115	−0.072	−0.1	0.146	0.046	−0.052	0.186
Fusobacterium	−0.142	0.032	−0.114	−0.132	−0.049	0.018	0.006
Massilia	−0.066	−0.248	−0.098	−0.197	−0.215	0.259	0.094
Cutibacterium	0.113	−0.22	−0.167	−0.219	−0.298*	0.178	0.09
Ralstonia	0.209	−0.326*	−0.08	−0.098	−0.163	0.282*	0.148
Veillonella	−0.135	0.084	−0.105	−0.139	−0.131	0.097	0.204
Mobiluncus	0.092	0.002	−0.098	0.073	0.084	0.144	0.289*
Parvimonas	0.049	0.162	−0.095	0.216	0.247	0.05	0.132
Murdochiella	0.249	−0.153	0.083	0.241	0.282*	−0.063	0.012
Acinetobacter	0.09	0.055	−0.051	−0.214	−0.266*	0.234	0.201
Gemella	−0.117	−0.037	−0.126	0.038	0.031	0.211	0.308*
Porphyromonas	0.064	−0.044	0.023	0.017	−0.127	−0.137	0.157
Brevundimonas	−0.280*	−0.079	−0.365**	−0.136	−0.292*	0.135	0.274*
Paracoccus	0.025	0.107	−0.115	−0.174	−0.115	0.224	0.013
Gardnerella	0.02	−0.062	0.045	−0.092	0.012	−0.046	0.178
Clostridium	0.084	0.058	0.028	0.16	0.139	0.003	0.022
Actinomyces	0.142	−0.11	0.061	0.021	−0.116	0.2	0.146
Deinococcus	−0.045	0.027	0.176	0.17	0.179	−0.151	0.002
Bacillus	0.165	−0.284*	0.058	0.192	0.001	0.087	0.032
Sphingobium	0.109	0.218	0.101	−0.029	0.071	0.119	0.084
Suicoccus	−0.024	−0.054	0.025	0.056	−0.061	0.046	0.005
Bradyrhizobium	0.269	−0.164	−0.104	−0.032	0.037	−0.061	−0.151
Bacteroides	0.131	−0.049	−0.085	0.054	−0.021	−0.133	0.147
Schaalia	−0.053	0.353**	0.086	0.19	0.203	−0.041	−0.146
Neisseria	−0.018	0.223	0.037	−0.101	−0.062	−0.021	−0.018
Paenibacillus	0.321*	−0.358**	−0.114	0.226	0.012	0.234	0.264*
Aerococcus	0.096	−0.144	0.206	−0.016	0.018	−0.2	−0.025
Actinotignum	−0.029	0.068	0.097	0.002	0.022	−0.048	−0.054
Flavobacterium	−0.059	−0.106	−0.015	−0.307*	−0.317*	−0.111	0.049
Sphingomonas	−0.054	0.281*	0.067	−0.312*	−0.296*	0.119	0.121
Cupriavidus	0.023	−0.156	0.001	−0.123	−0.04	**0**.378**	0.157
Fermentimonas	−0.06	0.094	−0.069	−0.112	−0.138	−0.101	0.179
Xylanimonas	0.242	−0.142	0.007	0.212	0.151	−0.181	−0.178
Janibacter	−0.033	−0.038	0.002	−0.05	−0.144	−0.139	0.129
Tepidanaerobacter	0.17	−0.03	0.066	0.156	0.272*	0.053	0.037
Bifidobacterium	0.14	−0.122	0.048	−0.081	−0.161	0.083	0.096
Kocuria	0.046	0.137	−0.02	−0.102	−0.154	−0.005	−0.042
Propionimicrobium	0.062	−0.053	0.1	0.161	0.092	−0.206	−0.026
Megasphaera	0.297*	−0.142	0.15	0.069	0.031	−0.07	−0.019
Steroidobacter	0.016	0.012	−0.011	−0.192	−0.143	0.172	−0.085
Brochothrix	−0.038	−0.076	−0.096	−0.045	0.075	−0.047	0.031
Rhodopseudomonas	0.310*	−0.08	−0.231	−0.241	−0.126	0.032	0.004
Haemophilus	0.169	0.078	0.077	0.053	0.11	0.036	0.114
Enterococcus	0.098	−0.036	−0.055	0.279*	0.137	0.229	0.288*
Oligotropha	0.279*	−0.056	−0.270*	−0.218	−0.11	0.025	0.017
Glaciecola	0.036	0.059	−0.061	0.021	0.069	−0.006	0.138
Filifactor	0.002	−0.017	0.023	0.132	−0.055	−0.079	0.151
Thermoanaerobacterium	0.101	0.107	0.056	0.214	0.206	0.131	0.139

Labeled in bold are the statistically significant correlations. *Statistically significant correlations (*p* < 0.05) ** Statistically significant correlations (*p* < 0.01).

Although the Alkaline assay and the TUNEL assay evaluate the presence of equivalent parameters (i.e. global DNA damage or fragmentation), the microbiome that correlates with one assay differs substantially from that of the other (Table 5). Higher abundances of the *Caulobacteraceae* family and its genus *Brevundimonas* was found in samples with lower levels of global sperm DNA fragmentation obtained by the TUNEL assay, as well as the *Oligotropha* genus. However, Alkaline Comet assay’s fragmentation levels was found to increase with higher abundance of *Thermoanaerobacteriaceae*, *Murdochiella, Tepidanaerobacter* and *Enterococcus*, and to decrease with higher abundance of the *Moraxellaceae* (specially *Moraxella* genus) and *Lactobacillaceae* (specially *Lactobacillus* genus) families and *Cutibacterium*, *Acinetobacter*, *Brevundimonas*, *Flavobacterium* and *Sphingomonas* genera. Finally, sperm double-stranded DNA fragmentation assessed by Neutral Comet assay was associated with increased abundance of the *Burkholderiaceae* family (and its genera *Ralstonia* and *Cupriavidus*), and with *Paenibacillaceae* (and *Paenibacillus* genus), *Enterococcaceae* (and *Enterococcus* genus), *Mobiluncus, Brevundimonas* and *Gemella*.

### Impact of Seminal Microbiota on Fertility

Regarding the presence/absence of bacteria, results obtained showed that the abundance of the *Phylobacteriaceae* and *Vibrionaceae* families was lower in seminal samples from donors than from infertile patients (*p* < 0.001 and *p* < 0.002, respectively). The use of these families for the prediction of infertility leads to an odds ratio of 0.052 (95% confidence interval [CI]: 0.005–0.364) with a sensitivity of 0.038 and a specificity of 0.567 in the case of *Phylobacteriaceae*, and an odds ratio of 0.122 (95% CI: 0.034–0.467) with a sensitivity of 0.094 and a specificity of 0.542 in the case of *Vibrionaceae*.

**FIGURE 4:**
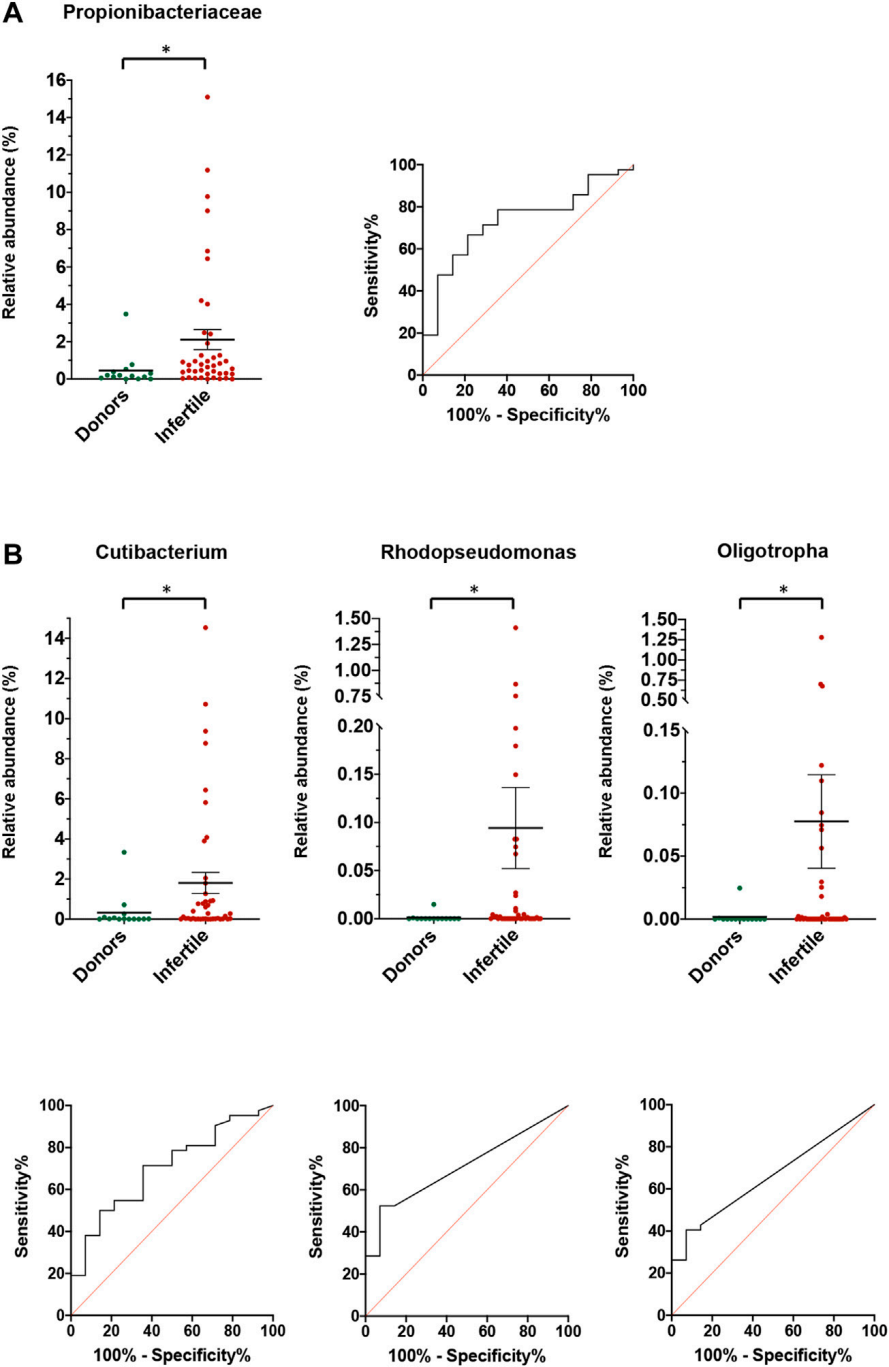
Relative abundance for donors and fertile individuals and infertile patients for **(A)** families, and **(B)** genera. For each family or genera, the relative abundance and the ROC curve for the prediction of infertility is shown. Lines and bars show average ±SEM. *Statistically significant differences between the two groups.

Comparing the relative abundance of all bacteria between control and infertile groups, results showed that the *Propionibacteriaceae* family presented differences between groups (*p =* 0.008). The evaluation of the abundance of this family as a predictor for fertility presented an area under the ROC curve (AUC) of 0.735 (95% CI: 0.593–0.876), with a cut-off value of 0.78% (Figure 4). Differences between control and infertile groups were also observed in the genera *Oligotropha* (*p =* 0.036) (AUC: 0.660; CI: 0.512 to 0.807; cut off value: 0.001%), *Rhodopseudomonas* (*p =* 0.008) (AUC: 0.711; CI: 0.573 to 0.849; cut off value: 0.001%) and *Cutibacterium* (*p =* 0.024) (AUC: 0.701; CI: 0.551–0.851; cut off value: 0.743%) (Figure 4).

In order to test if a combination of the three genera might lead to a higher predictive value for male infertility, a regression variable was generated through the Principal Component Analysis. The regression component was defined by the following formula:

Regression variable=0.999×Oligotropha+0.999×Rhodopseudomonas−0.031×Cutibacterium

The newly-generated regression variable depicted differences between control and infertile individuals (*p =* 0.001), an area under the curve of 0.780, CI: 0.650 to 0.909 for the cut-off value −0.287.

## Discussion

### Seminal Microbiota

Seminal microbiota has become a research topic of interest thanks to recent findings that link the presence of seminal dysbiosis to male infertility (Altmäe et al., 2019; Koedooder et al., 2019; Lundy et al., 2020; Farahani et al., 2021). However, the limited number of published studies still makes information regarding the seminal microbiome unclear and sometimes even contradictory, perhaps due to methodological variability. One of the factors that influences the information published so far is which *16S rRNA* hypervariable regions were sequenced. It was shown that each of the nine regions (V1-V9) provided different microbial identification resolution (Guo et al., 2013; Kerrigan et al., 2019; Sirichoat et al., 2020). To overcome this limitation, the present study is based on full-length *16S rRNA* gene amplification.

The microbiome composition of the samples analyzed in this work comprised the phyla *Firmicutes*, *Proteobacteria*, *Actinobacteria* and *Bacteroidetes*. This phylum-level composition and its relative abundance was consistent with previous studies (Mändar et al., 2017; Chen et al., 2018; Monteiro et al., 2018; Baud et al., 2019; Yang et al., 2020; Lundy et al., 2021; Okwelogu et al., 2021; Yao et al., 2021). When analyzing deeper into family and genus levels, *Streptococcus, Staphylococcus* and *Dialister* and its family *Veillonellaceae* were found as recurrent genera in our samples, in agreement with previous published works, (Hou et al., 2013; Weng et al., 2014; Mändar et al., 2017; Chen et al., 2018; Monteiro et al., 2018; Baud et al., 2019; Yang et al., 2020; Bukharin et al., 2021; Lundy et al., 2021; Yao et al., 2021). In the Proteobacteria phylum, the most relevant genera in our analysis were *Campylobacter*, *Moraxella* and *Ralstonia*. Of these, *Campylobacter* has been previously described as a member of seminal microbiome (Weng et al., 2014; Mändar et al., 2017; Chen et al., 2018; Baud et al., 2019). *Ralstonia* was detected in Chinese populations (Hou et al., 2013; Yao et al., 2021) and the abundance of *Moraxella* was highlighted only by Monteiro (Monteiro et al., 2018). *Corynebacterium* and *Prevotella*, the more representative genera of phyla *Actinobacteria* and *Bacteroidetes* respectively, are both among the most recurrent bacteria in the literature (Hou et al., 2013; Weng et al., 2014; Mändar et al., 2017; Chen et al., 2018; Monteiro et al., 2018; Baud et al., 2019; Amato et al., 2020; Yang et al., 2020; Bukharin et al., 2021; Lundy et al., 2021; Okwelogu et al., 2021; Yao et al., 2021).

However, other genera observed in our samples contrasted with the relative abundances found in previous reports. For the *Firmicutes* phylum, the three most abundant genera in our sample study, *Finegoldia*, *Peptoniphilus* and *Anaerococcus,* have also been described in several previous studies as highly abundant in seminal microbiota, although none of them has been previously characterized as the most abundant genera (Hou et al., 2013; Weng et al., 2014; Mändar et al., 2017; Monteiro et al., 2018; Baud et al., 2019; Yao et al., 2021). The relative abundance of *Lactobacillus*, found in most of our samples, is lower than the abundance found in previous works (Hou et al., 2013; Weng et al., 2014; Mändar et al., 2017; Chen et al., 2018; Baud et al., 2019; Amato et al., 2020; Yang et al., 2020; Okwelogu et al., 2021), a fact also reported by Monteiro *et al.* and Lundy *et al.* (Monteiro et al., 2018; Lundy et al., 2021) (Figure 1). Finally, to our knowledge this is the first time that the genus *Ezakiella* has been detected in seminal samples.

The genera identified so far in the seminal microbiome have a common characteristic: they all include bacteria that live in conditions of very low oxygen levels, whether they are strictly anaerobic, microaerophilic, or facultatively anaerobic (William B. Whitman, 2015). They are common in epithelial and mucous membranes, so can often be found on the skin or in the gastrointestinal and genitourinary tracts. Some have been described as opportunistic pathogens, and all except the genera *Campylobacter* and *Prevotella* are gram-positive. Some have been described in female genital microbiota, being the predominant taxonomic group in the vaginal tract *Lactobacillus*, although studies have also found *Prevotella*, *Dialister*, *Peptoniphilus*, *Anaerococcus*, *Ezakiella* and *Finegoldia* (Ravel et al., 2011; Gajer et al., 2012; Human Microbiome Project Consortium, 2012; Mändar et al., 2015; Diop et al., 2017; Koedooder et al., 2019).

In terms of phylum, family and genus with similar characteristics, the bacteria found in our samples can be structured in two profiles. One of the profiles presented a prevalence of *Firmicutes*, where *Finegoldia* predominated, while the other had similar frequencies of *Firmicutes* and *Proteobacteria,* with *Peptoniphilus* and *Campylobacter* predominant. However, neither cluster correlated with sperm quality parameters or clinical groups. Thus, two subgroups of individuals exist, in terms of microbiota that may be due to aspects other than fertility. Several studies point out that other bacterial communities inhabiting different organs are affected by diet (Bibbò et al., 2016; Kolodziejczyk et al., 2019; Barrientos-Durán et al., 2020; Dall’Asta et al., 2021). In addition, the fact that there are bacteria in semen which have also been observed in the vaginal tract could be evidence of a possible relationship between male and female microbiota, and would make a study of interactions between the two bacterial populations of a sexually active couple interesting, as suggested by Mändar *et al.* (2015). Cluster analyses carried out so far have observed some coincident profiles, such as those characterized by *Prevotella*- or *Lactobacillus*-enrichment (Hou et al., 2013; Weng et al., 2014; Baud et al., 2019), but none of them were observed in this study.

No differences in alpha diversity were observed between clusters or clinical groups in our cohort. The Mändar group reported higher species diversity in male reproductive disease, while the Chen group reported a reduction in diversity in azoospermic patients (Mändar et al., 2017; Chen et al., 2018). Despite this, not all authors conducted a study of bacterial diversity, so information is still limited.

### Impact of Seminal Microbiota on Sperm Quality Parameters

As shown by this study, and by previous studies, the most common bacterial phylum in seminal fluid is *Firmicutes* (Hou et al., 2013; Weng et al., 2014; Mändar et al., 2017; Chen et al., 2018; Monteiro et al., 2018; Baud et al., 2019; Amato et al., 2020; Yang et al., 2020; Bukharin et al., 2021; Lundy et al., 2021; Okwelogu et al., 2021; Yao et al., 2021). Some of the most representative taxa are good candidates to play a role in maintaining or affecting the patient’s fertility capacity. Our results show that the genus *Peptoniphilus*, for example, correlates with a decrease in progressive sperm motility, thus suggesting a negative impact on this sperm characteristic. We observed that the *Bacilli* group contains genera that present the strongest correlations with fertility traits: some genera of the *Bacillaceae* and the *Paenibacillaceae* families have been linked to poor sperm quality, with negative impact on morphology, concentration, and higher levels of double-stranded DNA fragmentation and oxidative stress. Reports on *Lactobacillus,* perhaps the most documented genus of the *Bacilli* group, show conflicting results regarding its role in seminal quality. Some authors have found a decrease in the amount of *Lactobacillus* in infertile men with sperm motility problems or seminal hyperviscosity (Weng et al., 2014; Mändar et al., 2017; Monteiro et al., 2018; Baud et al., 2019). In contrast, other authors have described an increased presence of this genus in asthenozoospermic and azoospermic patients (Hou et al., 2013; Chen et al., 2018; Yang et al., 2020). In our cohort, samples with a high relative abundance of these bacteria showed a reduction in global sperm DNA fragmentation, suggesting a possible protective effect of *Lactobacillus* on DNA integrity. The mechanism by which this genus could act as a DNA integrity protector is not known. It has been suggested that the production of short-chain fatty acids (SCFA) by *Lactobacillus* prevents sperm lipid peroxidation by ROS (Barbonetti et al., 2011; Markowiak-Kopeć and Śliżewska, 2020), and contributes to the seminal plasma antioxidant defense.

Another *Firmicutes* that negatively correlates with sperm DNA integrity is *Megasphaera*. The presence of this genus is increased in infertile individuals and also in samples with high oxidative stress. *Megasphaera* members are known to produce acyl-CoA dehydrogenases (DuPlessis et al., 1998) which could increase ROS levels in seminal plasma and the risk of sperm DNA damage. Finally, an increased presence of *Enterococcus* has been observed in samples with a high degree of global and double-stranded DNA fragmentation. Other research groups have linked the presence of *Enterococcus* in seminal plasma with high levels of sperm DNA damage, in bacteriospermia patients and *in vitro* studies (Zeyad et al., 2018; Duracka et al., 2019).

*Comamonadaceae*, *Megasphaera*, *Ralstonia and Schaalia* are the taxa that have shown a large negative correlation with seminal viscosity. These bacteria might benefit from a more liquid medium, which would allow for higher mobility and would made resource availability easier, giving them a proliferative advantage. Alternatively, some of these genera might act directly on the viscosity of the medium through the production of acids or hydrolase enzymes, which would degrade seminal fluid proteins and promote their liquefaction (Kershaw-Young et al., 2013; da Fonseca Junior et al., 2020; Rateb, 2021).

As for possible beneficial relationships between host and seminal microbiome, the *Proteobacteria* phylum correlates with better seminal and sperm parameters. The genus *Moraxella* and its family, largely represented in our samples, have been found in high proportions in samples of donors, and in samples with low levels of global DNA fragmentation and better sperm morphology. Beneficial effects of *Moraxella* have also been observed in both respiratory and intestinal tracts, where stable colonies of this genus have been associated with good health and its deregulation has been linked to pathogenic effects (Clemente et al., 2012; Biesbroek et al., 2014; Bosch et al., 2017; van den Munckhof et al., 2020).

According to our data, the family *Burkholderiaceae*, mainly represented by *Ralstonia*, is also associated with a favorable semen viscosity, sperm morphology and DNA protamination, suggesting a beneficial effect. In contrast, Yang and others identified an abundant presence of *Ralstonia* in asthenozoospermic patients (Yang et al., 2020), suggesting a detrimental relationship between this genus and sperm motility. More research needs to be done on the effects of this genus on male fertility.

Another *Proteobacteria* with potentially beneficial results is *Brevundimonas*, a genus that has been observed most abundantly in individuals with low oxidative stress, high progressive sperm motility and low levels of global DNA fragmentation. These parameters are all intrinsically related to each other and, as suggested by several authors, this could imply a cause-effect mechanism (Taha et al., 2012; Peluso et al., 2013; Ribas-Maynou and Benet, 2019). The catalase activity and carotenoid production observed in *Brevundimonas* could contribute to the reduction of seminal oxidative stress (Vancanneyt et al., 2015; Liu et al., 2020), through a decrease in cell membrane damage caused by lipid peroxidation. If this peroxidation occurs, DNA would be more exposed to oxidative stress, and extensive DNA damage would be generated. Moreover, the cell membrane might become damaged, affecting sperm viability. Similar results are found in *Flavobacterium,* the only bacterial genus in the phylum *Bacteroidetes* which, according to our analysis, has a potential relationship with fertility.

Finally, within the phylum *Actinobacteria*, *Gardnerella* and its family *Bifidobacteriaceae* are associated with better sperm motility, both total and progressive. The relationship between this taxon and sperm motility was also observed by Weng and others (Weng et al., 2014), and may be due to the *Bifidobacteriaceae* antioxidant effect (Valcarce et al., 2017; Valcarce et al., 2019). *Propionibacteriaceae* bacteria, although increased in infertile patients, has been shown to be associated with sperm motility and low levels of global DNA fragmentation. In contrast, *Schaalia* and *Mobiluncus*, from *Actinomycetaceae* family, appears to be at increased levels in patients with poor sperm chromatin protamination and high levels of double-stranded DNA fragmentation. In fact, *Actinomycetaceae* has been shown to produce isoprenoids (Kawasaki et al., 2003), molecules that cause DNA fragmentation and induce apoptosis in tumoral cells (Mo and Elson, 1999). More studies are needed to better determine the possibility of this effect on sperm cells.

### Impact of Seminal Microbiota on Fertility Prognosis

In order to assess whether the observed microbiota can predict male infertility and become a potential biomarker, the presence or absence and differential abundance of identified bacteria was analyzed. Our data suggest that the presence of the *Phylobacteriaceae* and *Vibrionaceae* families are more frequently associated with infertility. In addition, the proportion of the family *Propionibacteriaceae* was different between the control and patient groups, with a greater abundance in the latter at a significance below 0.01. Its predictive capacity has an AUC of 0.73, considering infertile those individuals who exceed the cut-off point of 0.78% relative abundance. We can also highlight three genera with similar characteristics: *Oligotropha* and *Rhodopseudomonas*, both with a cut-off value of 0.001%, and *Cutibacterium*, with a cut-off value of 0.74%. On their own, these bacteria may predict the fertility potential of an individual, but their low cut-off values suggest that utility may be limited. For this reason, a combination of the three genera’s abundances in a principal component analysis was expected to open the way for predictive models based on multiple specific bacterial profiles. Accordingly, in our analyses, the AUC value for such a combination was superior to those for the separate genera.

## Conclusion

Seminal microbiota analysis based on the amplification of the full-length *16S rRNA* gene is a methodology that allows the identification of the most abundant bacterial groups in seminal plasma. In agreement with previous studies, seminal microbiota found in this study consisted mainly of bacteria belonging to four phyla: *Firmicutes, Proteobacteria, Actinobacteria* and *Bacteroidetes*. The most abundant bacterial genera found were *Finegoldia*, *Peptoniphilus*, *Anaerococcus*, *Campylobacter*, *Streptococcus*, *Staphylococcus*, *Moraxella*, *Prevotella*, *Corynebacterium* and *Lactobacillus*. This methodology also allowed us to identify genera not previously described in the seminal microbiome, such as *Ezakiella*, a component in vaginal microbiota.

Our findings and those of other research groups provide evidence for the role of seminal microbiota in male fertility. Despite having a low biomass, some of these bacteria show a consistent relationship with their metabolic or enzymatic activity, as well as correlations with several interrelated parameters, such as oxidative stress, DNA fragmentation and sperm motility. The mechanisms by which they interfere with these seminal and spermatic parameters are still unknown in most cases, but may be related to their metabolism or enzymatic activities. Further studies are needed to determine the true effect of each of the identified bacteria and the possible clinical applications that may improve semen quality and pregnancy expectations.

## Data Availability

The datasets presented in this study can be found in online repositories. The names of the repository/repositories and accession number(s) can be found in the article/Supplementary Material.
