# Supplementary material for: Seminal Microbiota of Idiopathic Infertile Patients and Its Relationship With Sperm DNA Integrity
Source: Front Cell Dev Biol. 2022 Jun 28;10:937157. doi: 10.3389/fcell.2022.937157 (PMC9275566; doi:10.3389/fcell.2022.937157)
Supplement: Supplementary file 1 [file DataSheet1.ZIP › Supplementary Figure 1.docx]

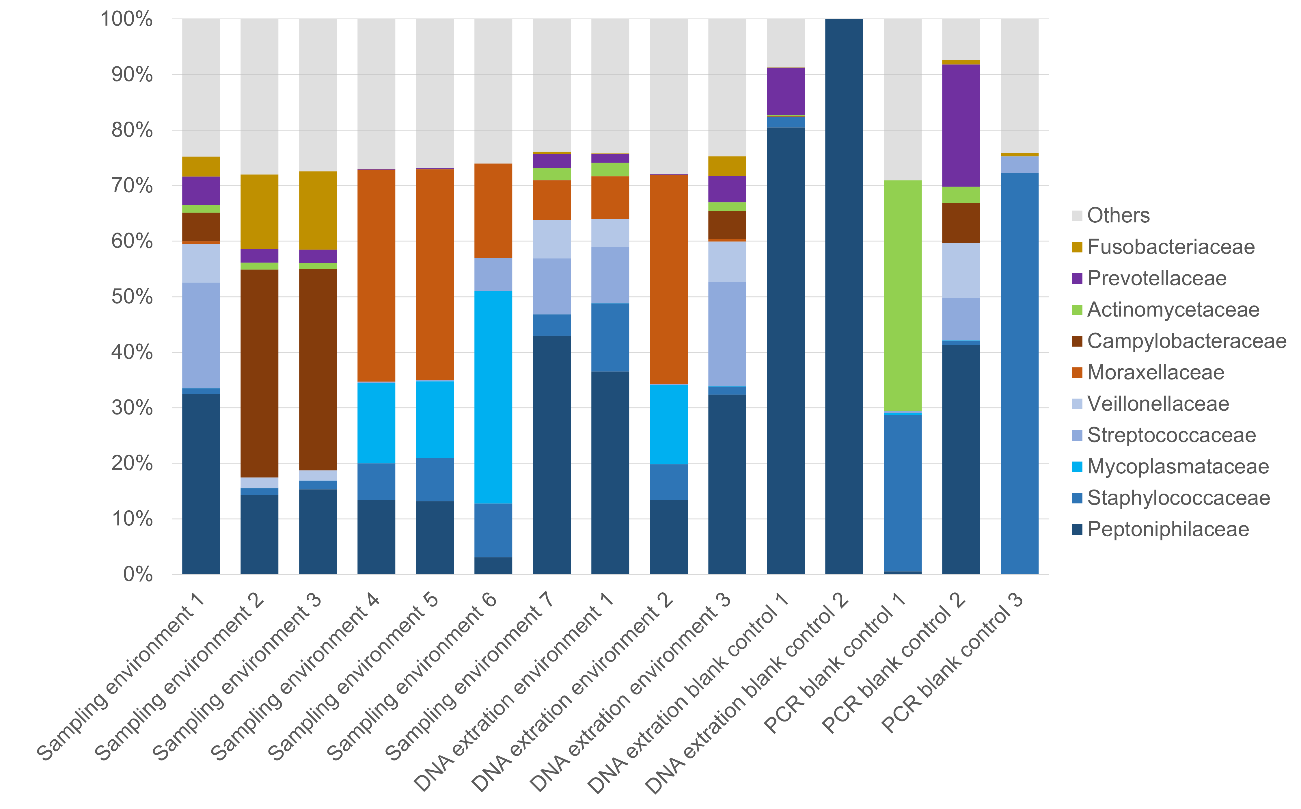


**Supplementary Figure 1.** Relative abundance in percentage of bacteria detected in negative controls at family taxonomic level. The X-axis shows each type of negative samples: environmental samples taken from the sample collection room (Sampling environment) and the laminar flux cabin where DNA extraction was processed (DNA extraction environment); and blank controls from DNA extraction (DNA extraction blank control) and PCR (PCR blank control) procedures to observe the *kitoma*.
