# Supplementary material for: Seminal Microbiota of Idiopathic Infertile Patients and Its Relationship With Sperm DNA Integrity
Source: Front Cell Dev Biol. 2022 Jun 28;10:937157. doi: 10.3389/fcell.2022.937157 (PMC9275566; doi:10.3389/fcell.2022.937157)
Supplement: Supplementary file 1 [file DataSheet1.ZIP › Supplementary table 1.docx]

**Supplementary table 1.** Demographic, lifestyle and health information of collected samples.

| **Clinic group** | **Age** | **Smoker** | **Alcohol intake** | **Caffeine (mg/day)** | **Radiation exposure** | **Chemical exposure** | **Antibiotic therapy*** | **Other diseases** |
| --- | --- | --- | --- | --- | --- | --- | --- | --- |
| Infertile | 40 | No | Sporadic | 388 | No | No | None | None |
| Infertile | 42 | No | Frequent | 156 | No | No | None | None |
| Infertile | 38 | -- | -- | -- | Yes | Yes | Yes | None |
| Infertile | 48 | -- | -- | -- | No | No | None | None |
| Infertile | 39 | No | Never | 0 | No | No | None | None |
| Infertile | 40 | -- | -- | -- | No | No | Yes | None |
| Infertile | 34 | Yes | Sporadic | 464 | No | No | None | Asthma |
| Infertile | 47 | Yes | Sporadic | 369 | No | No | None | Congenital heart disease |
| Infertile | 46 | Yes | Sporadic | 268 | No | Yes | None | Asthma, hiatal hernia |
| Infertile | 38 | Yes | Frequent | 268 | No | No |  | None |
| Infertile | 40 | No | Frequent | 348 | No | No | None | None |
| Infertile | 40 | No | Frequent | 355 | No | No | Yes | None |
| Infertile | 45 | No | Sporadic | 384 | No | No | Yes | None |
| Infertile | 41 | No | Sporadic | 116 | No | No | None | None |
| Infertile | 39 | No | Frequent | 384 | No | No | None | Bladder polyp, kidney stone |
| Infertile | 46 | No | Frequent | 348 | No | No | None | Disc hernia |
| Infertile | 34 | No | Sporadic | 123 | No | No | None | None |
| Infertile | 47 | No | Sporadic | 123 | No | No | Yes | None |
| Infertile | 38 | No | Sporadic | 572 | No | No | Yes | Inguinal hernia |
| Infertile | 36 | -- | -- | -- | No | No | None | None |
| Infertile | 36 | -- | -- | -- | No | Yes | None | None |
| Infertile | 41 | No | Frequent | 239 | No | No | Yes | None |
| Infertile | 39 | No | Sporadic | 47 | No | No | None | Hyperthyroidism |
| Infertile | 40 | No | Frequent | 7 | No | No | None | Circadian rhythm alteration |
| Infertile | 37 | No | Sporadic | 123 | No | No | None | None |
| Infertile | 40 | Yes | Never | 456 | No | No | None | None |
| Infertile | 42 | No | Sporadic | 464 | No | No | None | Urethral stenosis |
| Infertile | 39 | No | Frequent | 14 | No | No | None | None |
| Infertile | 43 | No | Sporadic | 471 | No | No | None | Hyperthyroidism |
| Infertile | 26 | No | Sporadic | 196 | No | No | Yes | None |
| Infertile | 42 | No | Frequent | 420 | No | Yes | None | None |
| Infertile | 39 | No | Sporadic | 152 | No | No | None | None |
| Infertile | 37 | No | Frequent | 116 | Yes | No | Yes | None |
| Infertile | 41 | No | Sporadic | 7 | No | No | None | None |
| Infertile | 28 | No | Never | 171 | No | Yes | Yes | Fistula |
| Infertile | 46 | No | Never | 348 | No | No | None | None |
| Infertile | 37 | No | Frequent | 239 | No | No | None | None |
| Infertile | 41 | No | Sporadic | 348 | No | Yes | None | None |
| Infertile | 37 | No | Never | 108 | No | No | None | None |
| Infertile | 47 | Yes | Frequent | 0 | No | No | None | Hypertension |
| Infertile | 32 | No | Never | 36 | No | No | None | None |
| Infertile | 36 | No | Never | 0 | No | No | None | Vas deferens cysts |
| Donor | 27 | No | Sporadic | 0 | No | No | None | None |
| Donor | 32 | No | Sporadic | 40 | No | No | None | None |
| Donor | 41 | No | Never | 7 | No | No | None | None |
| Donor | 27 | No | Sporadic | 464 | No | No | None | None |
| Donor | 26 | No | Sporadic | 116 | No | No | None | None |
| Donor | 27 | No | Frequent | 188 | No | No | Yes | None |
| Donor | 27 | No | Never | 159 | No | No | None | None |
| Donor | 33 | No | Sporadic | 159 | No | No | None | None |
| Donor | 48 | No | Sporadic | 239 | No | No | None | None |
| Donor | 25 | No | Frequent | 232 | No | No | Yes | None |
| Donor | 37 | No | Frequent | 55 | No | No | None | None |
| Donor | 32 | No | Sporadic | 239 | No | No | None | None |
| Donor | 40 | No | Never | 464 | No | No | None | None |
| Donor | 30 | Yes | Frequent | 239 | No | No | None | None |

*Antibiotic therapy during the last year. If yes, last dose of antibiotic taken at least 21 days before sample collection.
