# Supplementary material for: Seminal Microbiota of Idiopathic Infertile Patients and Its Relationship With Sperm DNA Integrity
Source: Front Cell Dev Biol. 2022 Jun 28;10:937157. doi: 10.3389/fcell.2022.937157 (PMC9275566; doi:10.3389/fcell.2022.937157)
Supplement: Supplementary file 1 [file DataSheet1.ZIP › Supplementary table 2.docx]

**Supplementary table 2**. Relative abundance of taxa identified by 16S rRNA sequencing in semen samples. The mean proportion of each taxon with a minimum relative abundance of 0.05% in whole sample is shown.

|  | **Total (%)** | **Controls (%)** | **Idiopathic patients (%)** |
| --- | --- | --- | --- |
| **Phylum** |  |  |  |
| Firmicutes | 58.66 | 60.89 | 57.92 |
| Proteobacteria | 19.06 | 19.38 | 18.96 |
| Actinobacteria | 8.00 | 5.49 | 8.84 |
| Bacteroidetes | 5.34 | 7.00 | 4.79 |
| Tenericutes | 2.25 | 2.05 | 2.32 |
| Fusobacteria | 1.71 | 0.05 | 1.35 |
| Deinococcus Thermus | 0.24 | 0.00 | 0.32 |
| Spirochaetes | 0.07 | 0.05 | 0.08 |
| Saccharibacteria | 0.06 | 0.02 | 0.07 |
|  |  |  |  |
| **Family** |  |  |  |
| Peptoniphilaceae | 32.10 | 36.57 | 30.61 |
| Campylobacteraceae | 5.81 | 6.83 | 5.47 |
| Streptococcaceae | 4.80 | 5.61 | 4.53 |
| Moraxellaceae | 4.12 | 5.49 | 3.67 |
| Staphylococcaceae | 3.83 | 3.48 | 3.94 |
| Prevotellaceae | 3.79 | 5.45 | 3.23 |
| Corynebacteriaceae | 3.27 | 2.54 | 3.52 |
| Peptostreptococcaceae | 2.89 | 2.30 | 3.09 |
| Lactobacillaceae | 2.61 | 1.03 | 3.14 |
| Veillonellaceae | 2.61 | 2.75 | 2.56 |
| Mycoplasmataceae | 2.24 | 2.05 | 2.31 |
| Oxalobacteraceae | 2.15 | 2.92 | 1.90 |
| Propionibacteriaceae | 1.70 | 0.45 | 2.11 |
| Fusobacteriaceae | 1.65 | 2.73 | 1.29 |
| Actinomycetaceae | 1.32 | 1.40 | 1.29 |
| Burkholderiaceae | 1.13 | 1.01 | 1.16 |
| Bradyrhizobiaceae | 0.73 | 0.09 | 0.94 |
| Rhodobacteraceae | 0.57 | 0.51 | 0.59 |
| Caulobacteraceae | 0.45 | 0.22 | 0.53 |
| Bacillaceae | 0.44 | 0.32 | 0.48 |
| Porphyromonadaceae | 0.43 | 0.34 | 0.46 |
| Sphingomonadaceae | 0.43 | 0.43 | 0.43 |
| Bifidobacteriaceae | 0.37 | 0.13 | 0.46 |
| Aerococcaceae | 0.35 | 0.29 | 0.37 |
| Clostridiaceae | 0.29 | 0.16 | 0.33 |
| Flavobacteriaceae | 0.27 | 0.26 | 0.28 |
| Neisseriaceae | 0.26 | 0.05 | 0.33 |
| Deinococcaceae | 0.24 | 0.00 | 0.32 |
| Paenibacillaceae | 0.22 | 0.04 | 0.28 |
| Bacteroidaceae | 0.18 | 0.21 | 0.17 |
| Micrococcaceae | 0.18 | 0.03 | 0.23 |
| Lachnospiraceae | 0.18 | 0.08 | 0.21 |
| Comamonadaceae | 0.17 | 0.00 | 0.23 |
| Enterobacteriaceae | 0.17 | 0.02 | 0.22 |
| Intrasporangiaceae | 0.13 | 0.00 | 0.17 |
| Thermoanaerobacteraceae | 0.10 | 0.08 | 0.11 |
| Pasteurellaceae | 0.10 | 0.03 | 0.12 |
| Promicromonosporaceae | 0.10 | 0.17 | 0.07 |
| Methylobacteriaceae | 0.09 | 0.20 | 0.05 |
| Listeriaceae | 0.09 | 0.01 | 0.11 |
| Erysipelotrichaceae | 0.08 | 0.07 | 0.09 |
| Hungateiclostridiaceae | 0.08 | 0.10 | 0.07 |
| Tissierellaceae | 0.08 | 0.10 | 0.07 |
| Steroidobacteraceae | 0.08 | 0.01 | 0.10 |
| Acidiferrobacteraceae | 0.08 | 0.01 | 0.10 |
| Erwiniaceae | 0.07 | 0.02 | 0.09 |
| Enterococcaceae | 0.07 | 0.02 | 0.08 |
| Spirochaetaceae | 0.07 | 0.05 | 0.07 |
| Sinobacteraceae | 0.06 | 0.03 | 0.08 |
| Alteromonadaceae | 0.06 | 0.09 | 0.05 |
| Thermoanaerobacterales | 0.06 | 0.06 | 0.06 |
| Leptotrichiaceae | 0.06 | 0.05 | 0.06 |
| Atopobiaceae | 0.06 | 0.06 | 0.06 |
| Piscirickettsiaceae | 0.06 | 0.00 | 0.07 |
|  |  |  |  |
| **Genera** |  |  |  |
| Finegoldia | 13.03 | 14.14 | 12.66 |
| Peptoniphilus | 11.13 | 13.48 | 10.35 |
| Anaerococcus | 6.97 | 7.76 | 6.71 |
| Campylobacter | 5.78 | 6.78 | 5.45 |
| Streptococcus | 4.75 | 5.57 | 4.48 |
| Staphylococcus | 3.80 | 3.47 | 3.90 |
| Moraxella | 3.71 | 5.11 | 3.24 |
| Prevotella | 3.58 | 5.14 | 3.06 |
| Ezakiella | 3.06 | 4.05 | 2.74 |
| Corynebacterium | 3.04 | 2.28 | 3.29 |
| Lactobacillus | 2.60 | 1.02 | 3.12 |
| Ureaplasma | 2.23 | 2.04 | 2.30 |
| Dialister | 1.69 | 2.13 | 1.55 |
| Fusobacterium | 1.64 | 2.72 | 1.29 |
| Massilia | 1.52 | 2.02 | 1.35 |
| Cutibacterium | 1.44 | 0.33 | 1.81 |
| Ralstonia | 0.74 | 0.49 | 0.82 |
| Veillonella | 0.67 | 0.41 | 0.76 |
| Mobiluncus | 0.44 | 0.41 | 0.45 |
| Parvimonas | 0.40 | 0.40 | 0.40 |
| Murdochiella | 0.40 | 0.61 | 0.32 |
| Acinetobacter | 0.33 | 0.31 | 0.34 |
| Gemella | 0.32 | 0.15 | 0.38 |
| Porphyromonas | 0.31 | 0.24 | 0.34 |
| Brevundimonas | 0.30 | 0.16 | 0.35 |
| Paracoccus | 0.27 | 0.21 | 0.29 |
| Gardnerella | 0.27 | 0.04 | 0.34 |
| Clostridium | 0.26 | 0.13 | 0.31 |
| Actinomyces | 0.26 | 0.29 | 0.24 |
| Deinococcus | 0.24 | 0.00 | 0.32 |
| Bacillus | 0.23 | 0.15 | 0.25 |
| Sphingobium | 0.21 | 0.20 | 0.21 |
| Suicoccus | 0.21 | 0.13 | 0.23 |
| Bradyrhizobium | 0.18 | 0.02 | 0.23 |
| Bacteroides | 0.18 | 0.21 | 0.17 |
| Schaalia | 0.17 | 0.16 | 0.17 |
| Neisseria | 0.16 | 0.03 | 0.20 |
| Paenibacillus | 0.16 | 0.03 | 0.20 |
| Aerococcus | 0.14 | 0.16 | 0.14 |
| Actinotignum | 0.14 | 0.20 | 0.12 |
| Flavobacterium | 0.14 | 0.17 | 0.13 |
| Sphingomonas | 0.13 | 0.15 | 0.12 |
| Cupriavidus | 0.11 | 0.31 | 0.04 |
| Fermentimonas | 0.10 | 0.09 | 0.10 |
| Xylanimonas | 0.10 | 0.17 | 0.07 |
| Janibacter | 0.09 | 0.00 | 0.12 |
| Tepidanaerobacter | 0.09 | 0.08 | 0.10 |
| Bifidobacterium | 0.08 | 0.08 | 0.09 |
| Kocuria | 0.08 | 0.01 | 0.11 |
| Propionimicrobium | 0.08 | 0.03 | 0.10 |
| Megasphaera | 0.08 | 0.04 | 0.09 |
| Steroidobacter | 0.08 | 0.01 | 0.10 |
| Brochothrix | 0.07 | 0.01 | 0.10 |
| Rhodopseudomonas | 0.07 | 0.00 | 0.09 |
| Haemophilus | 0.07 | 0.02 | 0.09 |
| Enterococcus | 0.06 | 0.02 | 0.08 |
| Oligotropha | 0.06 | 0.00 | 0.08 |
| Glaciecola | 0.06 | 0.09 | 0.05 |
| Filifactor | 0.06 | 0.02 | 0.07 |
| Thermoanaerobacterium | 0.06 | 0.05 | 0.06 |
| Spirochaeta | 0.05 | 0.04 | 0.06 |
| Thiomicrospira | 0.05 | 0.00 | 0.07 |
| Sinimarinibacterium | 0.05 | 0.01 | 0.07 |
