# Supplementary material for: Seminal Microbiota of Idiopathic Infertile Patients and Its Relationship With Sperm DNA Integrity
Source: Front Cell Dev Biol. 2022 Jun 28;10:937157. doi: 10.3389/fcell.2022.937157 (PMC9275566; doi:10.3389/fcell.2022.937157)
Supplement: Supplementary file 1 [file DataSheet1.ZIP › Supplementary table 3.docx]

**Supplementary table 3**. Clustering analysis results for (A) phylum, (B) family and (C) genus taxonomic levels. The mean relative abundance and the 95% confidence interval (CI) of each taxon in each cluster is shown.

| **A *PHYLUM*** | **Cluster 1**  Relative abundance mean (95% CI) | **Cluster 2**  Relative abundance mean (95% CI) |
| --- | --- | --- |
| *Firmicutes* | 65.83 (61.98 – 69.67) | 39.09 (28.42 – 49.76) |
| *Proteobacteria* | 14.58 (11.9 – 17.26) | 31.31 (19.77 – 42.85) |
| *Actinobacteria* | 8.24 (6.23 – 10.25) | 7.36 (3.47 – 11.25) |
| *Tenericutes* | 0.02 (0.01 – 0.03) | 8.34 (1.73 – 14.95) |
| *Bacteroidetes* | 6.37 (3.61 – 9.13) | 2.54 (0.62 – 4.46) |
| *Saccharibacteria* | 0.03 (0.00 – 0.06) | 0.12 (0.00 – 0.36) |
| *Deinococcus Thermus* | 0 (0.00 – 0.01) | 0.9 (0.00 – 2.82) |
| *Fusobacteria* | 0.31 (0.06 – 0.56) | 5.53 (1.24 – 9.81) |
| *Spirochaetes* | 0.09 (0.05 – 0.12) | 0.02 (0.00 – 0.04) |
|  |  |  |
| **B *FAMILY*** | **Cluster 1**  Relative abundance mean (95% CI) | **Cluster 2**  Relative abundance mean (95% CI) |
| *Peptoniphilaceae* | 35.19 (28.50 – 41.89) | 27.98 (18.85 – 37.11) |
| *Campylobacteraceae* | 9.55 (5.72 – 13.37) | 0.84 (0.00 – 1.74) |
| *Streptococcaceae* | 4.47 (1.93 – 7.01) | 5.24 (2.32 – 8.16) |
| *Moraxellaceae* | 1.38 (0.31 – 2.45) | 7.78 (2.57 – 12.99) |
| *Staphylococcaceae* | 0.79 (0.35 – 1.22) | 7.88 (4.15 – 11.61) |
| *Prevotellaceae* | 6.34 (3.74 – 8.94) | 0.38 (0.09 – 0.68) |
| *Corynebacteriaceae* | 1.63 (0.50 – 2.75) | 5.47 (3.29 – 7.65) |
| *Peptostreptococcaceae* | 4.65 (2.64 – 6.66) | 0.55 (0.05 – 1.06) |
| *Lactobacillaceae* | 1.24 (0.01 – 2.46) | 4.44 (0.00 – 10.20) |
| *Veillonellaceae* | 4.12 (3.00 – 5.24) | 0.60 (0.05 – 1.14) |
| *Mycoplasmataceae* | 0.02 (0.01 – 0.03) | 5.21 (0.92 – 9.51) |
| *Oxalobacteraceae* | 0.33 (0.14 – 0.51) | 4.59 (2.75 – 6.44) |
| *Propionibacteriaceae* | 0.75 (0.33 – 1.16) | 2.96 (1.15 – 4.78) |
| *Fusobacteriaceae* | 2.72 (0.65 – 4.80) | 0.22 (0.00 – 0.45) |
| *Actinomycetaceae* | 1.91 (1.34 – 2.48) | 0.52 (0.11 – 0.92) |
| *Burkholderiaceae* | 0.27 (0.08 – 0.45) | 2.27 (0.62 – 3.92) |
|  |  |  |
| **C *GENUS*** | **Cluster 1**  Relative abundance mean (95% CI) | **Cluster 2**  Relative abundance mean (95% CI) |
| *Finegoldia* | 14.88 (10.32 – 19.44) | 9.94 (6.51 – 13.37) |
| *Peptoniphilus* | 8.55 (5.66 – 11.43) | 15.45 (10.82 – 20.07) |
| *Anaerococcus* | 6.96 (4.96 – 8.95) | 7.00 (2.66 – 11.33) |
| *Campylobacter* | 1.66 (0.65 – 2.68) | 12.65 (7.36 – 17.93) |
| *Streptococcus* | 5.77 (3.21 – 8.33) | 3.04 (0.55 – 5.54) |
| *Staphylococcus* | 5.84 (3.13 – 8.56) | 0.39 (0.20 – 0.57) |
| *Moraxella* | 5.93 (2.49 – 9.36) | 0.01 (0.00 – 0.02) |
| *Prevotella* | 0.76 (0.30 – 1.22) | 8.28 (4.91 – 11.65) |
| *Ezakiella* | 0.41 (0.07 – 0.76) | 7.48 (3.64 – 11.32) |
| *Corynebacterium* | 4.55 (2.89 – 6.21) | 0.52 (0.19 – 0.86) |
| *Lactobacillus* | 3.09 (0.00 – 7.00) | 1.79 (0.00 – 3.66) |
| *Ureaplasma* | 3.57 (0.58 – 6.56) | 0.00 (0.00 – 0.01) |
| *Dialister* | 0.19 (0.07 – 0.31) | 4.19 (2.90 – 5.49) |
| *Fusobacterium* | 0.27 (0.03 – 0.51) | 3.93 (0.84 – 7.02) |
| *Massilia* | 2.38 (1.34 – 3.42) | 0.09 (0.01 – 0.18) |
| *Cutibacterium* | 2.28 (1.04 – 3.52) | 0.03 (0.01 – 0.04) |
