# Supplementary figures and images for: Seminal Microbiota of Idiopathic Infertile Patients and Its Relationship With Sperm DNA Integrity
Source: Front Cell Dev Biol. 2022 Jun 28;10:937157. doi: 10.3389/fcell.2022.937157 (PMC9275566; doi:10.3389/fcell.2022.937157)

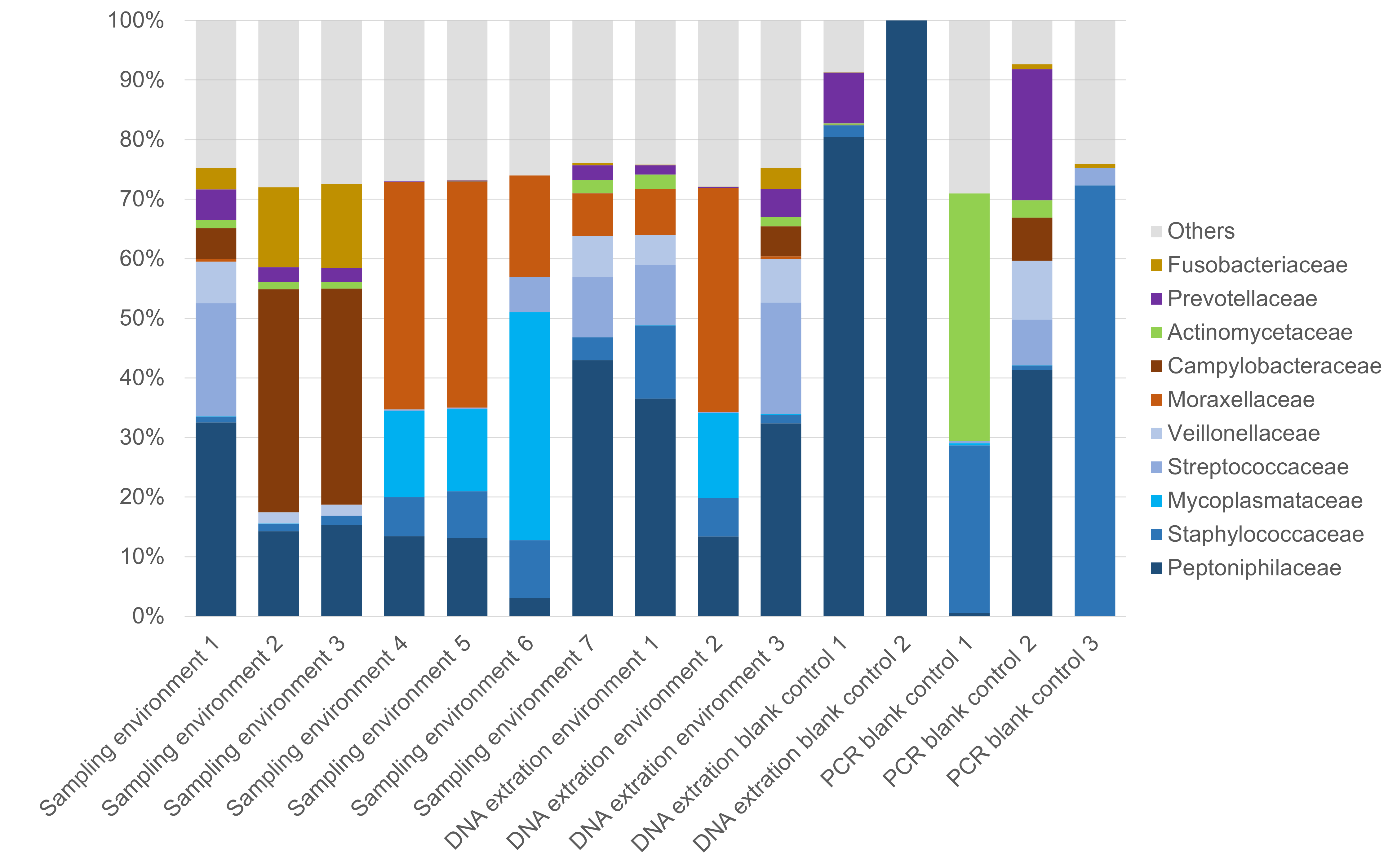

Supplement: Supplementary file 1 [file DataSheet1.ZIP › Supplementary Figure 1.tiff]
